# From malnutrition to multimodal care: a bibliometric and knowledge mapping analysis of frailty research in cancer

**DOI:** 10.3389/fnut.2026.1732736

**Published:** 2026-05-15

**Authors:** Xiaoxia Gao, Wenjun Pan, Yifeng Wang, Mengjing Xu

**Affiliations:** 1 The 2^nd^ Affiliated Hospital and Yuying Children’s Hospital of Wenzhou Medical University/The 2^nd^ School of Medicine, Wenzhou Medical University, Wenzhou, Zhejiang, China; 2 Municipal Hospital Affiliated to Taizhou University, Taizhou, Zhejiang, China

**Keywords:** cholesterol blood level, liver disease, lymph node metastasis, mini nutritional assessment short-form (MNA-SF), short physical performance battery (SPPB)

## Abstract

**Background:**

Frailty and malnutrition are highly prevalent, interrelated syndromes in cancer that significantly impact treatment tolerance, clinical outcomes, and quality of life. Despite rapid growth in research over the past two decades, the structure, evolution, and emerging frontiers of this field remain unclear. This study presents a comprehensive bibliometric and knowledge-mapping analysis of global research on frailty and nutrition in cancer, using data from the Web of Science Core Collection (WoSCC) and Scopus databases, to identify major contributors, research hotspots, and future trends.

**Methods:**

Publications related to frailty and nutrition in cancer from 2005 to 2025 were retrieved from WoSCC and Scopus on September 30, 2025. Bibliometric and knowledge mapping were analyzed using CiteSpace, VOSviewer, and bibliometrix.

**Results:**

A total of 2,887 publications were identified, showing a substantial surge in output after 2020, with projections indicating a peak around 2032. The field is currently dominated by research in oncology, biochemistry, and rehabilitation disciplines. Recent studies emphasize the development of standardized diagnostic criteria, mechanistic insights, and multimodal management strategies. Keyword burst and trend analyses identified several emerging frontiers including cholesterol blood level, lymph node metastasis, short physical performance battery (SPPB), liver disease, and mini nutritional assessment short-form (MNA-SF).

**Conclusion:**

Global research on frailty and nutrition in cancer has evolved from descriptive epidemiology to evidence-based, precision, and integrative frameworks combining clinical nutrition, geriatric oncology, and rehabilitation science. Current research hotspots emphasize prehabilitation, multimodal interventions, and the integration of functional, metabolic, and imaging markers into personalized care. Future directions should focus on developing predictive models, digital health-enabled assessments, and multicenter translational collaborations to improve early detection, optimize interventions, and inform patient-centered, policy-driven cancer care strategies.

## 1 Introduction

Malignant tumors pose one of the most significant global public health challenges. According to GLOBOCAN 2020 data from the International Agency for Research on Cancer (IARC), an estimated 19.29 million new cancer cases and 9.96 million cancer-related deaths occurred worldwide in 2020 (1). With the global population aging rapidly and advancements in cancer therapies, survival rates for cancer patients have markedly improved. However, frailty and malnutrition have emerged as critical clinical challenges across the cancer care continuum, severely affecting treatment tolerance, long-term outcomes, and quality of life (2, 3). Frailty is a multidimensional geriatric syndrome characterized by reduced physiological reserve, multisystem dysregulation, and decreased resistance to stressors, making individuals more vulnerable to disease, surgery, and treatment-related toxicity (2). It is increasingly recognized as an independent prognostic factor in oncology: frail cancer patients exhibit significantly shorter overall survival (OS) and progression-free survival (PFS), with a 1.5 to 3-fold higher mortality risk compared to non-frail patients (4). While frailty affects approximately 10 to 15% of the general elderly population, it prevalence rises sharply to 25 to 50% among cancer patients, exceeding 60% in older adults undergoing chemotherapy (4). Malnutrition another major concern in oncology, affects 30 to 70% of cancer patients, often coexisting with frailty, though the two conditions only partially overlap (5). Emerging evidence suggests that malnutrition may accelerate the development of frailty through mechanisms such as chronic inflammation, metabolic dysregulation, and inadequate energy intake, while frailty worsens anorexia, digestive dysfunction, and nutrient malabsorption, creating a vicious cycle (5, 6). Therefore, early detection and effective management of both conditions are critical to improving survival and functional outcomes in cancer care.

With the rise of precision medicine and personalized care, the assessment and management of frailty and nutritional status in cancer patients have garnered increasing attention. Their pathophysiology is multifactorial, involving tumor burden, treatment toxicity, chronic inflammation, metabolic disorders, and sarcopenia (7, 8). Shared biological pathways, including systemic inflammation, oxidative stress, metabolic imbalance, and endocrine alterations are believed to underlie both conditions by accelerating muscle catabolism and physiological decline (9, 10). Key risk factors include advanced age, low body mass index (BMI), hypoalbuminemia, poor functional capacity, and multimorbidity (11). A range of tools are currently utilized to screen for and assess these conditions. Common frailty assessments include the Fried phenotype, Frailty Index, and Eastern Cooperative Oncology Group Performance Status (ECOG-PS), while nutritional screening typically involves the Patient-Generated Subjective Global Assessment (PG-SGA) and Nutritional Risk Screening 2002 (NRS-2002) (12, 13). In the oncological context, standardized malnutrition assessment tools have become increasingly important for identifying vulnerable patients and guiding supportive care strategies. Recent studies have highlighted the clinical utility of structured nutritional assessment instruments in improving risk stratification and informing individualized treatment planning in cancer populations (14–16). In addition to traditional screening methods, consensus-based diagnostic criteria such as the Global Leadership Initiative on Malnutrition (GLIM) framework have been increasingly adopted to improve the consistency and comparability of malnutrition diagnosis across clinical settings (17). Nutritional interventions have evolved from traditional dietary counseling to include enteral and parenteral nutrition, immunonutrition, and combined exercise-nutrition programs, signaling a shift toward multimodal, individualized approaches (18, 19). Research in this area has progressed from descriptive and correlational studies on nutritional status and clinical outcomes to a more mechanistic and integrative focus, encompassing biomarker discovery, body composition imaging, and multimodal interventional trials. However, several key questions remain unanswered, including the optimal timing for frailty screening, standardized criteria for nutritional assessment, appropriate intervention strategies for different tumor types, and the development of predictive prognostic models.

Despite growing scholarly interest, most existing studies have concentrated on specific cancer types, singular nutritional indicators, or isolated clinical interventions. To date, no comprehensive bibliometric study has mapped the global research landscape on frailty and nutrition in cancer. Bibliometric analysis, a data-driven approach that combines quantitative science mapping and network visualization provides a robust framework for systematically characterizing research trends, intellectual structures, and emerging frontiers within a scientific domain (20). By analyzing publication trajectories, citation networks, authorship patterns, institutional collaborations, keyword co-occurrence, reference clustering, and citation bursts, bibliometrics offers a quantitative and visual understanding of the evolution of scientific knowledge. This method has been extensively utilized in medical and health sciences to inform research strategies, funding priorities, and clinical policy development. Existing bibliometric analyses on frailty in cancer have largely focused on the epidemiology of frailty and sarcopenia, the impact of frailty on surgical outcomes and prognosis, and the role of comprehensive geriatric assessment (CGA) in identifying vulnerable patients (21–24). However, no bibliometric study to date has specifically addressed the intersection of frailty and malnutrition in cancer patients. Thus, this study aims to perform a comprehensive bibliometric and knowledge mapping analysis of global research on frailty and nutrition in cancer, using integrated data from both the Web of Science Core Collection (WoSCC) and Scopus databases. By examining temporal publication trends, citation influence, and collaboration networks, and by applying keyword co-occurrence and reference clustering analyses, this study seeks to clarify the knowledge structure, research hotspots, and emerging frontiers in this field. The findings will offer valuable insights for researchers, clinicians, and policymakers, facilitating future research planning, strategic resource allocation, and the development of evidence-based approaches to the prevention and management of frailty and malnutrition in cancer care.

## 2 Materials and methods

### 2.1 Data sources and search strategy

The WoSCC and Scopus databases were widely recognized sources for bibliometric analysis. To ensure the comprehensiveness and representativeness of the dataset, this study retrieved literature from both the WoSCC and Scopus databases related to frailty, nutrition, and cancer. The search strategy was based on a thorough review of previous literature and MeSH terms, search keywords were refined through multiple pilot tests and expert validation. The final search string used in WoSCC was as follows: TS = (“Frailt*” OR “Frailness” OR “Frailty Syndrome” OR “Sarcopenia*”) AND TS = (“Tumor*” OR “Neoplasia*” OR “Neoplasm*” OR “Cancer*” OR “Malignant Neoplasm*” OR “Malignanc*”) AND TS = (“Nutrition* Assessment*” OR “Nutrition* Index*” OR “Nutrition* Indices” OR “Prognostic Nutritional Index” OR “Prognostic Nutritional Indices” OR “Mini Nutrition* Assessment*” OR “Parenteral Nutrition Solutions” OR “Intravenous Feeding Solutions” OR “Nutrition* Status” OR “Malnutrition” OR “Malnourishment*” OR “Undernutrition” OR “Nutritional Deficienc*” OR “Protein-Energy Malnutrition*” OR “Protein-Calorie Malnutrition” OR “Marasmus”). For Scopus, an equivalent query was applied to the title, abstract, and keywords fields.

**Figure 1:**
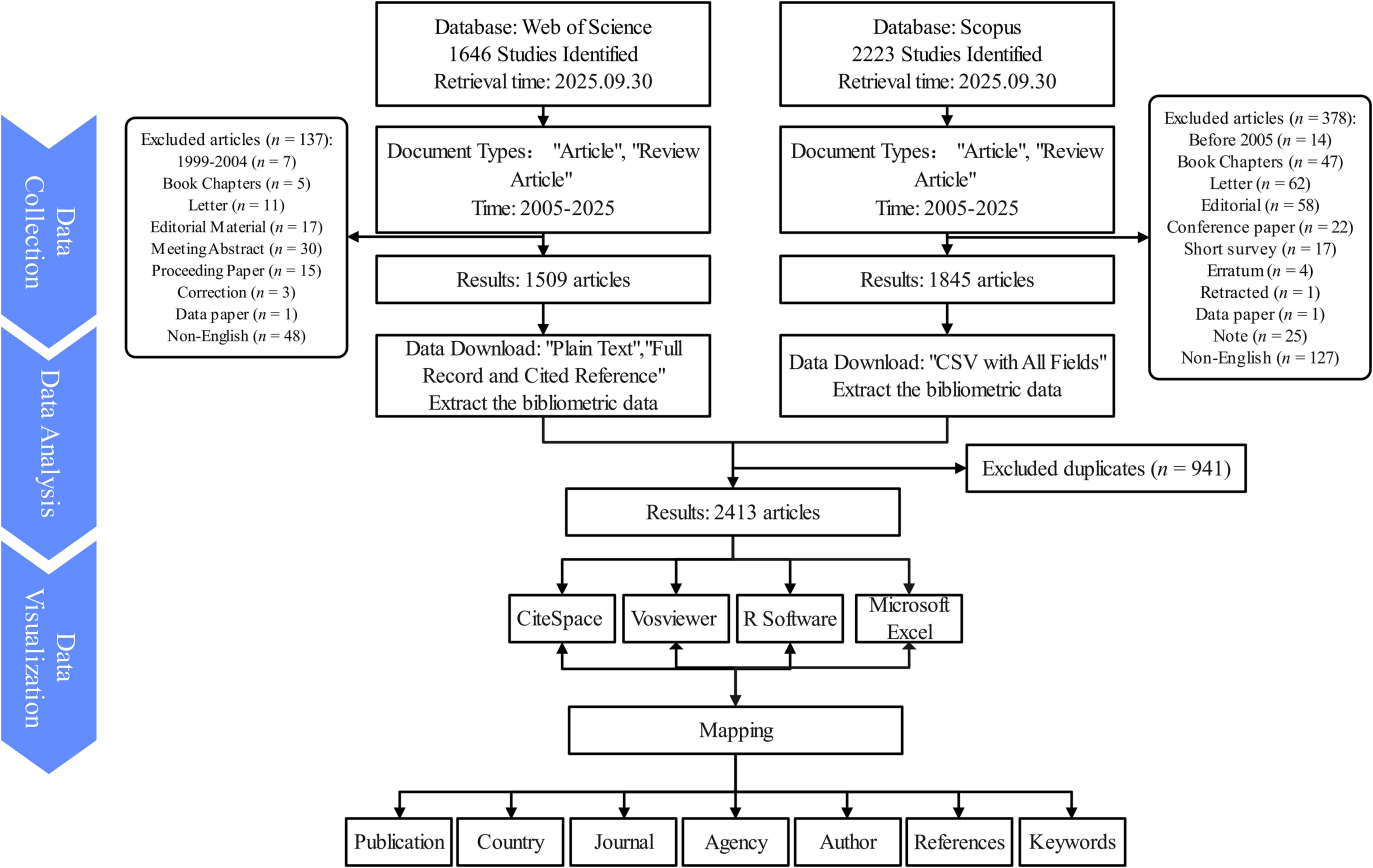
Overview of the research design and literature screening process in the bibliometric study of nutrition and frailty in cancer. This flowchart illustrates the data sources, inclusion and exclusion criteria, final dataset selection, and key phases of bibliometric analysis, including co-authorship, keyword clustering, and co-citation analysis. It visually summarizes the methodological framework and analytical workflow used in the study.

To maintain data accuracy, all searches, data extraction, and downloads in both databases were conducted independently by two researchers on September 30, 2025. Any discrepancies were resolved through discussion with a third senior researcher. In the WoSCC, 1,646 records were initially retrieved, and after restricting publication years to 2005–2025, limiting document types to articles and reviews, and excluding meeting abstracts, editorials, letters, and notes, 1,509 eligible documents were retained. The same filters were applied in the Scopus database 1,845 records were yielded. All WoSCC records were exported in Plain Text (Full Record and Cited References) format, while Scopus data were exported in CSV (All Fields) format (Figure 1). Since the coverage of WoSCC and Scopus partially overlaps but is not identical, the two datasets were merged and cleaned to eliminate duplicates. The deduplication process followed a standardized protocol based on DOI, title, and first author. Journal names, author affiliations, and country names were normalized to resolve database inconsistencies. After removing 941 duplicates, a total of 2,413 unique records were included for analysis. In cases of duplicate records between databases, the WoSCC record was preferentially retained because its metadata are more compatible with bibliometric software. All subsequent analyses were performed on the merged dataset to maximize representativeness and minimize database bias.

### 2.2 Data analysis

Bibliometric and visualization analyses were performed using VOSviewer 1.6.20, CiteSpace 6.4. R1, Bibliometrix (R package), R software, and Microsoft Excel 2019.

#### 2.2.1 Bibliometrix

Bibliometrix is an open-source R package developed by Massimo Aria and Corrado Cuccurullo (25), and is one of the most widely recognized tools for bibliometric and science mapping analysis. It enables quantitative assessment of annual publication trends, author and institutional productivity, co-authorship and co-citation networks, and thematic evolution.

#### 2.2.2 CiteSpace

CiteSpace is a Java-based bibliometric visualization software widely applied in scientific knowledge mapping (26–28). It was used in this study to analyze the structural (e.g., betweenness centrality), temporal (e.g., citation bursts), and evolutionary dimensions of the field. Node size reflects frequency, and link thickness represents the strength of co-occurrence or co-citation. Parameter settings were as follows: Years per slice: 1 year, Selection criteria: g-index (k = 25).

#### 2.2.3 VOSviewer

VOSviewer is another widely recognized software for constructing and visualizing bibliometric networks (29). It was employed to create author collaboration and keyword co-occurrence maps, helping to identify major contributors, thematic clusters, and interrelationships among research topics.

## 3 Results

### 3.1 Annual publication output and growth trend

**Figure 2:**
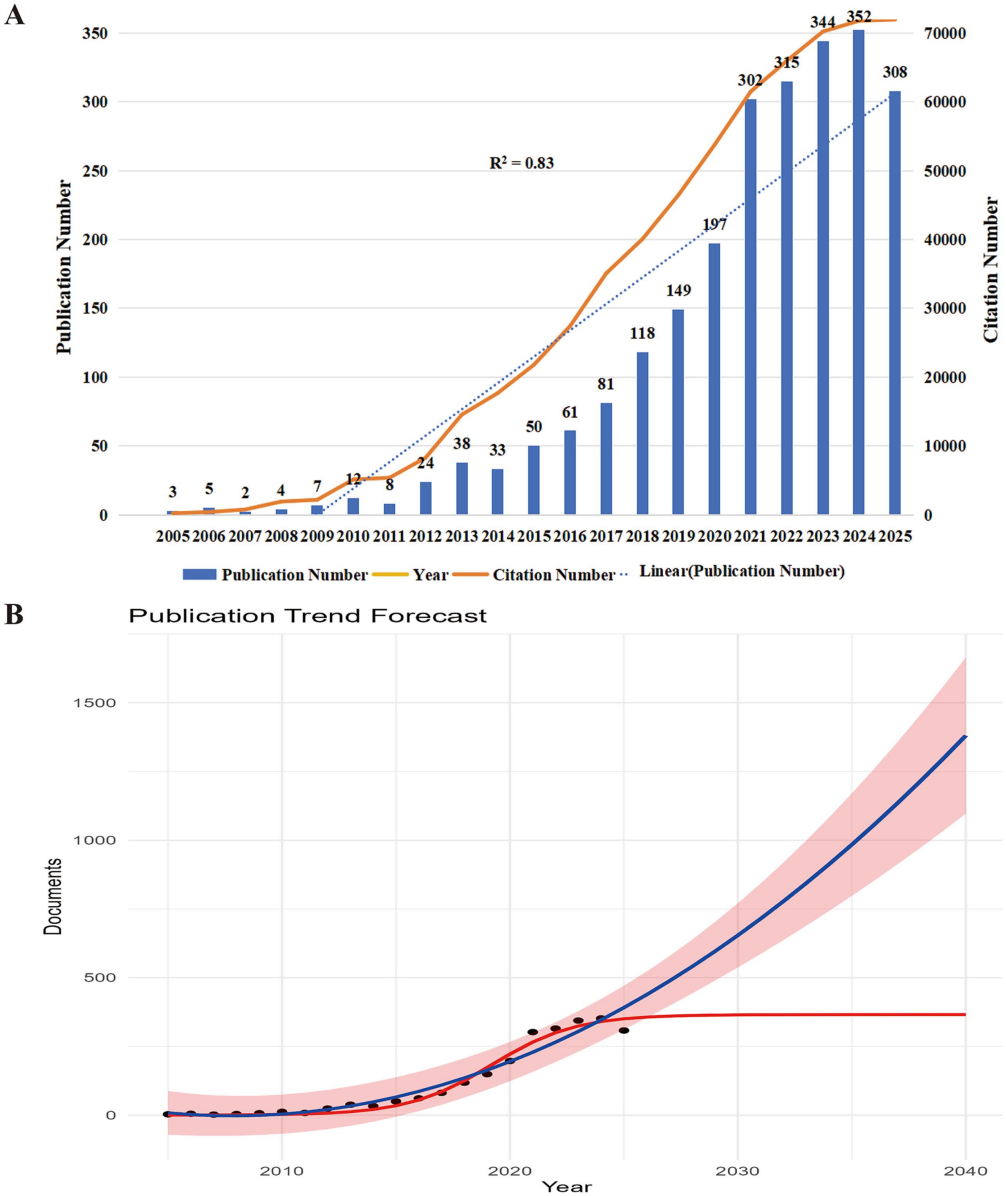
Temporal trends and future projection of research output on nutrition and frailty in cancer. **(A)** Annual number of publications (bars) and citations (line) from 2005 to 2025. **(B)** Trend prediction of annual publications (2025–2040). Black dots represent actual publication counts from 2005 to 2025, while the red curve illustrates the projected annual publication trend. The prediction indicates a steady increase, suggesting sustained and expanding research interest in this field.

Analyzing annual publication output and growth trends offers valuable insights into the global research activity and academic influence within the field (27). On September 30, 2025, a total of 2,413 publications on frailty and nutrition in cancer were retrieved from WoSCC and Scopus databases, contributed by 10,290 authors across 141 countries and published in 728 journals. Each paper involved an average of 7.53 authors, and the international collaboration rate reached 9.24%. As shown in Figure 2A, the number of publications has grown rapidly, from only 2 papers in 2007 to 352 papers in 2024, representing an approximately 180-fold increase. The annual growth rate was 26.06%, with a strong upward trajectory (R^2^ = 0.83). Publication output began to rise notably after 2012 and accelerated sharply after 2020. The average document age was 3.89 years, indicating that research in this domain is relatively recent and increasingly recognized as a critical component of cancer care.

Forecasting the publication trends for 2025 and the next decade (Figure 2B) revealed that the number of publications would reach 351 in 2025, with a projected peak of approximately 366 papers around 2032, suggesting that research on frailty and nutrition in cancer patients will continue to attract sustained academic attention in the coming years.

### 3.2 Country analysis

**Figure 3:**
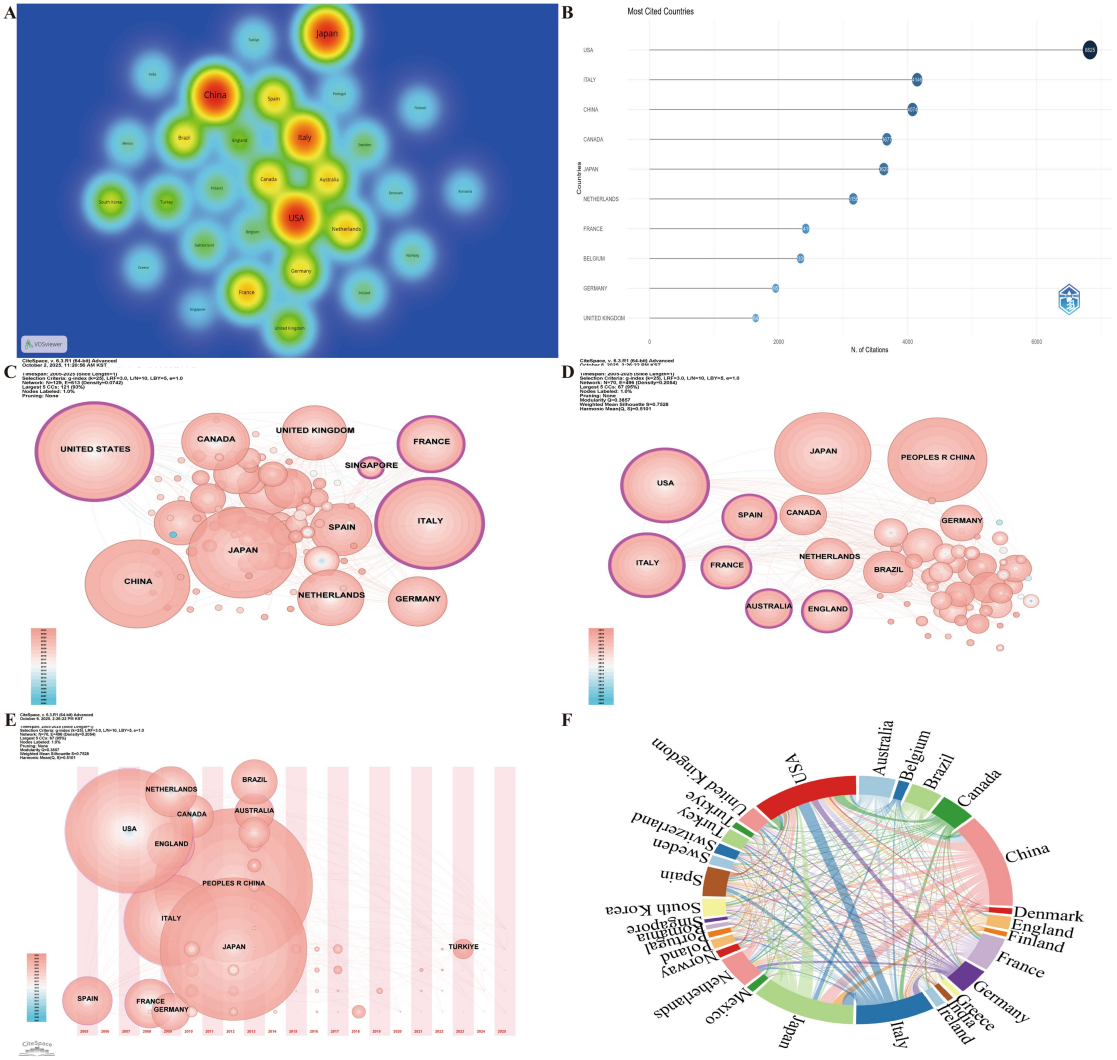
Global research distribution and international collaboration on nutrition and frailty in patients with cancer. **(A)** Global heatmap of publication output by country. **(B)** Top 10 most-cited countries. **(C)** Betweenness centrality of countries in the Scopus dataset. Node size corresponds to the frequency of co-occurrence, while nodes encircled in purple indicate high centrality (≥ 0.1), signifying influential bridging roles. **(D)** Betweenness centrality of countries in the WoSCC dataset. **(E)** Co-occurrence network of contributing countries by publication timezone. The horizontal axis displays the year of each country’s initial publication in frailty and nutritional status research. Node size represents the publication volume, with blue nodes indicating earlier contributions and pink nodes signifying recent publications. Overlapping colors indicate multiple publications within a single year, forming rings that reflect consistent and extensive publication activity over time, demonstrating each country’s ongoing engagement in the field. **(F)** International collaboration network among the top 30 most productive countries, with link thickness representing the strength of inter-country collaboration.

Country analysis helps identify the most influential nations in the field and reveals patterns of international collaboration. A total of 141 countries contributed publications related to frailty and nutrition in cancer patients. As shown in Figure 3A, the top 10 countries by publication volume were: China (269), Japan (231), USA (154), Italy (113), Korea (74), Brazil (30), France (31), Netherlands (32), Spain (33), and the United Kingdom (34). In terms of total citations, the top 10 countries were the USA (6,825), Italy (4,146), China (4,074), Canada (3,677), Japan (3,629), Netherlands (3,156), France (2,419), Belgium (2,339), Germany (1,953), and the UK (1,643; Figure 3B). CiteSpace betweenness centrality analysis further revealed that the USA, France, and Italy play pivotal roles and exert significant influence in this field (Figures 3C,D). The country timezone view illustrated that Spain was among the earliest countries to publish studies in this area and has maintained consistent research output since then (Figure 3E). Collaboration network analysis among the top 30 countries (≥20 publications) revealed that the USA and Italy partnership was the strongest, followed by China and Japan (Figure 3F). However, the overall international collaboration rate (9.24%) remains relatively low, indicating that cross-national cooperation still has considerable space for improvement.

### 3.3 Institutional analysis

**Figure 4:**
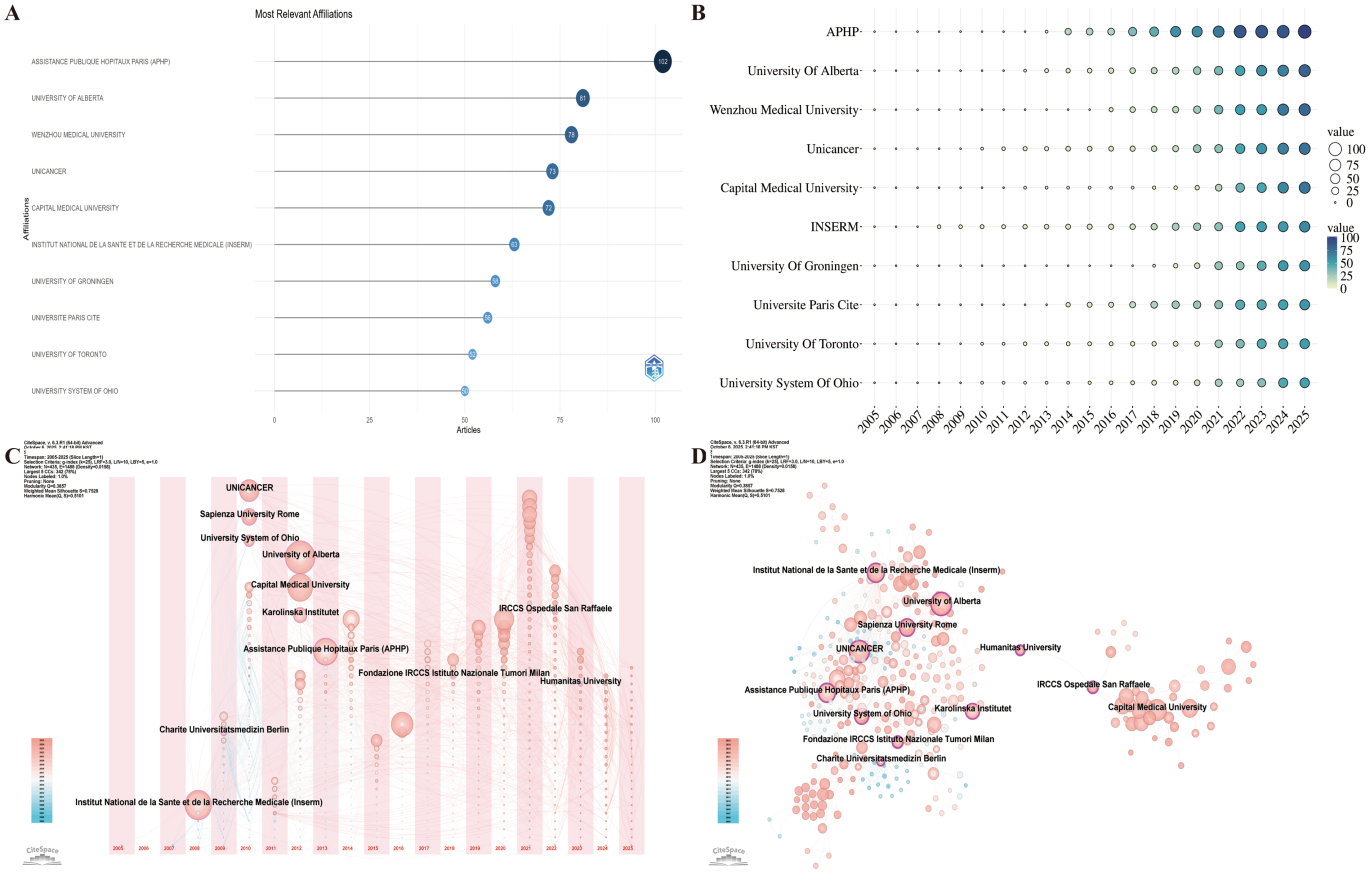
Institutional productivity and collaboration network in research on nutrition and frailty in patients with cancer. **(A)** Top 10 most productive institutions. **(B)** Annual publication trends of the top institutions. **(C)** Co-occurrence network showing the timezone of contributing institutions. **(D)** Institutional betweenness centrality network showing inter-institutional collaboration and influence.

Institutional analysis identifies the most productive research institutions and their collaboration networks, offering valuable insights for future academic exchanges. A total of 434 institutions contributed to this field. The top 10 most productive institutions were: Assistance Publique Hopitaux Paris (APHP; 102), University of Alberta (81), Wenzhou Medical University (78), Unicancer (73), Capital Medical University (72), Institut National de la Santé et de la Recherche Médicale (INSERM) (31), University of Groningen (32), Université Paris Cité (35), University of Toronto (33), and University System of Ohio (36) (Figure 4A). INSERM was identified as one of the earliest institutions to publish in this field (since 2008), as shown in Figure 4B, consistent with the result of institutional timezone view (Figure 4C). Centrality analysis based on the WoSCC dataset identified 12 institutions with strong bridging roles in the collaboration network (Figure 4D), including Fondazione IRCCS Istituto Nazionale Tumori Milan (0.27), Humanitas University (0.25), APHP (0.19), Sapienza University of Rome (0.17), University of Alberta (0.16), Charite Universitatsmedizin Berlin (0.16), Karolinska Institutet (0.15), University System of Ohio (0.12), INSERM (0.11), and UNICANCER (0.10). These institutions serve as core nodes driving international collaboration and academic innovation in this field.

### 3.4 Author analysis

**Figure 5:**
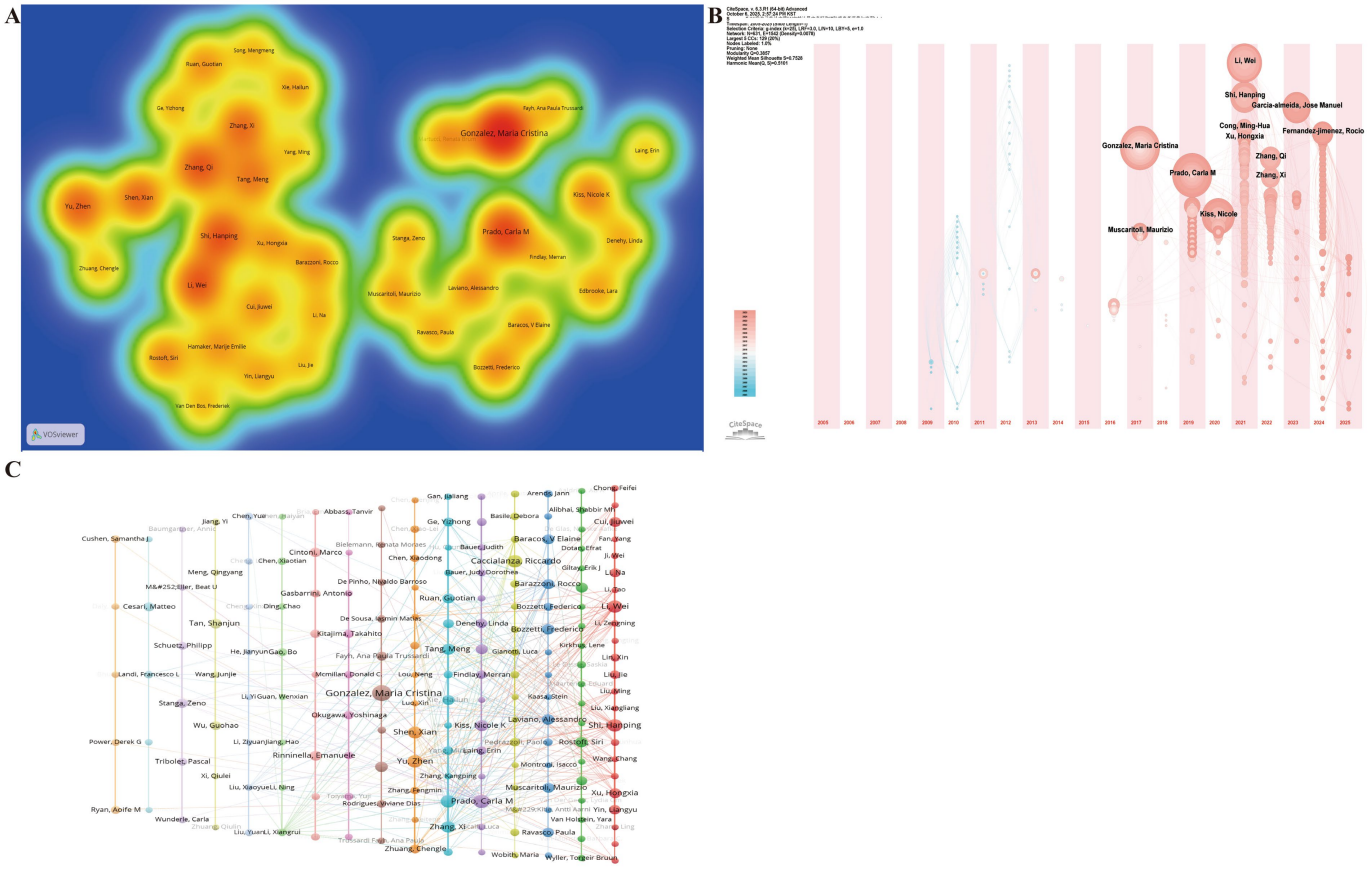
Author collaboration network in research on nutrition and frailty in patients with cancer. **(A)** Author co-occurrence heatmap. The intensity of the color represents the frequency of co-occurrence between authors, with red color indicating higher co-occurrence frequencies. **(B)** Author timezone showing the year of first publication and subsequent research activity. **(C)** Co-authorship clustering map illustrating major author clusters and their collaborative relationships.

Author analysis highlights the leading experts and core research teams, offering valuable insights for identifying collaboration opportunities and tracking research trends. A total of 10,290 authors contributed to the retrieved publications. The top 10 most productive authors were Prado, Carla M (28), Shi, Hanping (27), Li, Wei (19), Zhang, Qi (17), Kiss, Nicole K (16), Zhang, Xi (16), Liu, Jie (15), Xu, Hongxia (15), Tang, Meng (15), and Song, Chunhua (14) (Figure 5A). The author timezone view (Figure 5B) revealed that 2021 marked a notable increase in the number of new contributors who subsequently maintained continuous publication activity, consistent with the sharp rise in overall publication output observed in the same year (Figure 2A). Cluster analysis of the 419 authors with more than five publications identified 16 author clusters (Figure 5C). The largest cluster (red) comprised 23 Chinese authors, led by Shi Hanping and Li Wei; the second cluster (green) included 20 authors led by Rostoft, Siri; and the third cluster (blue) included 17 authors, led by Bozzetti, Frederico. These clusters reflect the formation of several geographically distinct but thematically cohesive research groups.

### 3.5 Journal analysis

**Figure 6:**
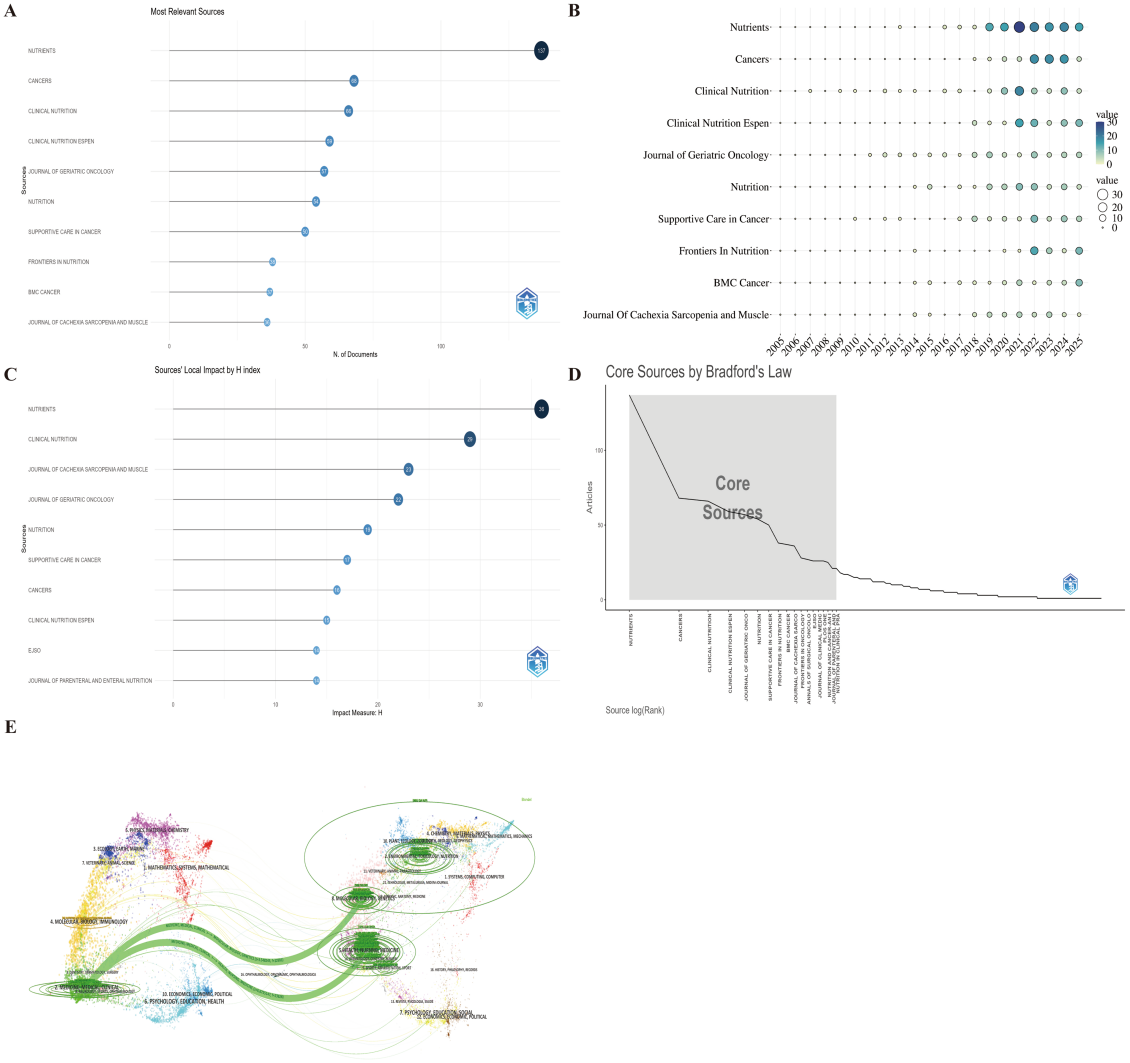
Journal-level analysis of publication and citation trends on nutrition and frailty in cancer patients. **(A)** Top 10 journals by publication count. **(B)** Annual publication output of the top 10 journals. **(C)** Top 10 journals ranked by local H-index. **(D)** Bradford’s law distribution identifying core journals. **(E)** Dual-map overlay, showing citation flows between citing (left) and cited (right) journal clusters. Colored paths represent cross-disciplinary knowledge transfer between research domains.

Journal analysis provides insights into the academic impact, trends, and research priorities, guiding researchers in journal selection and identifying emerging topics. A total of 728 journals published studies on frailty and nutrition in cancer. The top 10 most productive journals were: Nutrients (137), Cancers (37), Clinical Nutrition (30), Clinical Nutrition ESPEN (38), Journal of Geriatric Oncology (39), Nutrition (40), Supportive Care in Cancer (36), Frontiers in Nutrition (41), BMC Cancer (42), and Journal of Cachexia, Sarcopenia and Muscle (43) (Figure 6A). As shown in Figure 6B, Clinical Nutrition was among the earliest journals to publish research on this topic. According to local H-index rankings, the leading journals were Nutrients (43), Clinical Nutrition (29), Journal of Cachexia, Sarcopenia and Muscle (23), Journal of Geriatric Oncology (22), Nutrition (19), Supportive Care in Cancer (17), Cancers (16), Clinical Nutrition ESPEN (15), EJSO (14), and Journal of Parenteral and Enteral Nutrition (14) (Figure 6C), consistent with Bradford’s Law analysis (Figure 6D). Among these journals, five were classified as JCR Q1, three as Q2, and two as Q3, indicating generally high publication quality. Most publications appeared in journals within the fields of geriatrics, nutrition, and oncology, underscoring the multidisciplinary nature of this topic.

The dual-map overlay of journals (Figure 6E) illustrates the citation trajectories and disciplinary evolution of the field. Due to database compatibility, the overlay was generated using the WoSCC dataset. In the map, the left side represents the citing journals, and the right side represents the cited journals, while the curved colored lines indicate the flow of knowledge across disciplines. The analysis revealed a clear disciplinary shift, from “medicine, medical, and clinical” journals toward “health, nursing, medicine” and “molecular, biology, and genetics” domains, suggesting that research in this field has evolved from descriptive clinical studies to biologically driven and population-based health management frameworks.

### 3.6 Reference analysis

**Figure 7:**
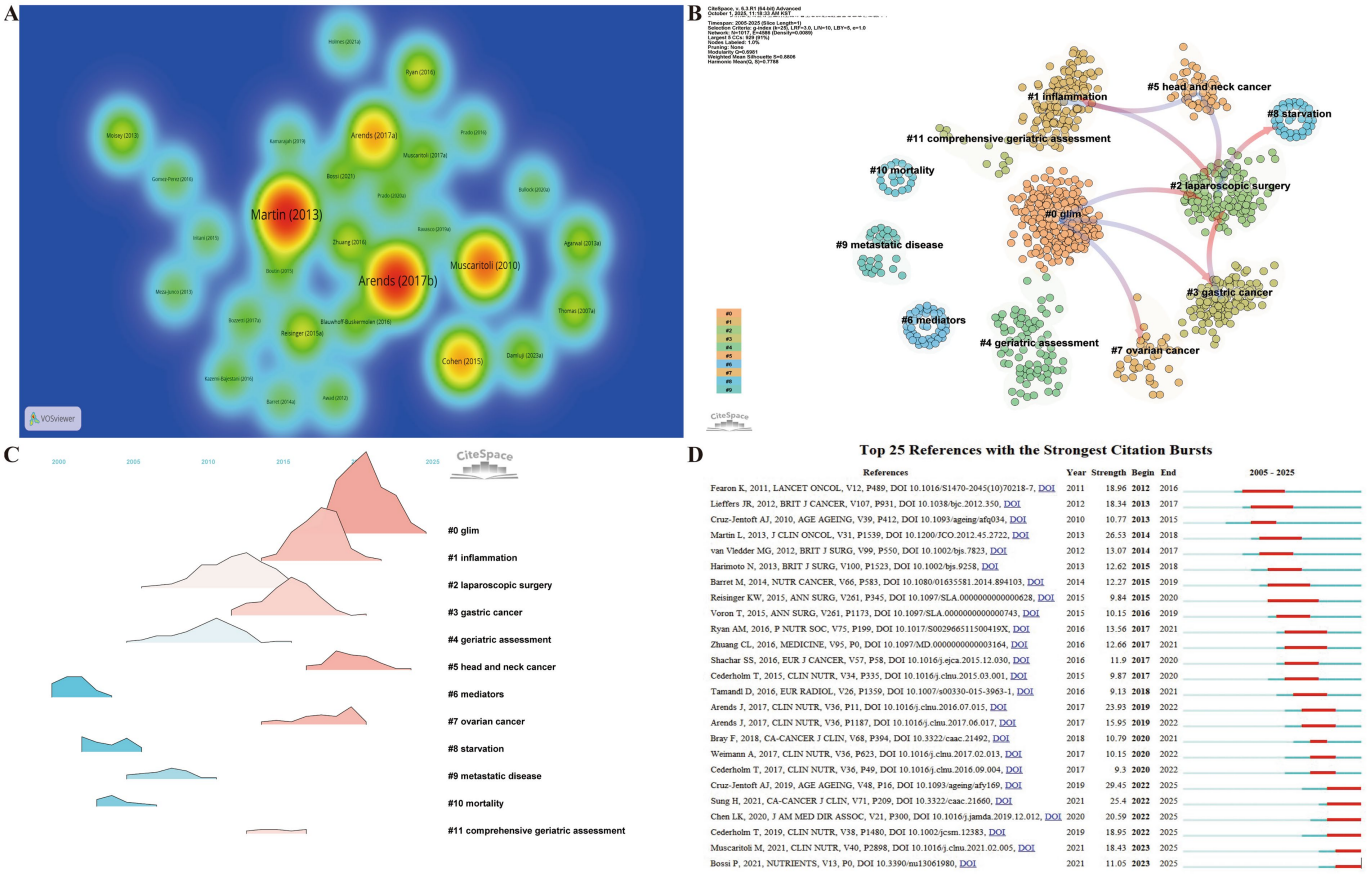
Co-citation and clustering analysis of references reveals major thematic domains and their temporal evolution. **(A)** Reference co-occurrence heatmap. **(B)** Reference cluster dependency map (WoSCC dataset). **(C)** Reference clustering landscape (WoSCC dataset). **(D)** Top 25 references with the strongest citation bursts (WoSCC dataset).

Reference analysis is crucial for uncovering the intellectual structure, research hotspots, interdisciplinary linkages, and academic influence within a field, offering insights into its historical development and guiding future directions (26). The top 10 most cited references (41–50) (Figure 7A) collectively define the intellectual foundation of research on frailty and nutrition in cancer. Among these, three studies focus on the core mechanisms of cancer-related malnutrition and frailty, three are international consensus statements and clinical guidelines, and four examine frailty assessment and nutritional interventions in older cancer patients. Together, these publications highlight a clear research trajectory—from mechanistic exploration to clinical application—and emphasize that standardized nutritional assessment and targeted interventions are essential strategies for mitigating frailty, enhancing resilience, and improving both quality of life and prognosis in cancer care. The ESPEN guidelines on nutrition in Cancer patients (2017) (42), which received the highest number of citations (*n* = 1,986), is a cornerstone document in oncology nutrition management. This guideline established an evidence-based framework for screening, assessment, and intervention in cancer-related malnutrition, formally linking nutritional care to frailty status, treatment tolerance, and patient outcomes.

Reference co-citation clustering not only identifies current research hotspots but also traces the evolutionary trajectory of a scientific field over time. Due to database limitations, separate analyses were conducted for the WoSCC and Scopus datasets. In the WoSCC dataset, 12 major clusters were identified (Figure 7B). The inter-cluster dependency analysis revealed the evolutionary relationships among these themes: pink arrows indicate foundational knowledge bases, while blue arrows denote emerging frontiers (Figure 7C). Cluster #0 (GLIM), focused on frailty assessment tools, evolved from clusters #2 (laparoscopic surgery), #3 (gastric cancer), and #7 (ovarian cancer), and remains active, highlighting the growing focus on standardized evaluation frameworks. Cluster #5 (head and neck cancer), addressing cancer cachexia and metabolic dysfunction, originated from clusters #1 (inflammation) and #2 (laparoscopic surgery), and continues to be highly active, highlighting the current research hotspots in the field.

Analysis of the top 25 references with the strongest citation bursts (Figure 7D) further emphasized emerging areas of focus. Six publications demonstrated ongoing citation bursts (1, 51–55), including four key international consensus and guideline documents—the EWGSOP2 (51), the updated AWGS consensus (52), the Global Leadership Initiative on Malnutrition (GLIM) criteria (53), and the ESPEN guidelines (54). These publications represent landmark contributions that collectively reshaped the diagnostic and management frameworks for frailty, sarcopenia, and malnutrition. The EWGSOP2 consensus (51) introduced a strength-based diagnostic algorithm for sarcopenia, shifting the focus from muscle mass quantification to functional performance. The Asian Working Group for Sarcopenia (AWGS) update (52) adapted these diagnostic standards for Asian populations and emphasized the significance of nutrition and exercise interventions. The GLIM criteria (53) established a two-step global diagnostic framework integrating both phenotypic and etiologic components of malnutrition. Building on these frameworks, the ESPEN guidelines (54) provided comprehensive, evidence-based recommendations for nutritional management across the cancer care continuum. The concurrent citation bursts of these publications signify the scientific community’s increasing acknowledgment of frailty and nutrition as interdependent factors in cancer outcomes. This transition also expanding the research focus from descriptive epidemiology to precision-oriented, evidence-based therapeutic frameworks.

**Figure 8:**
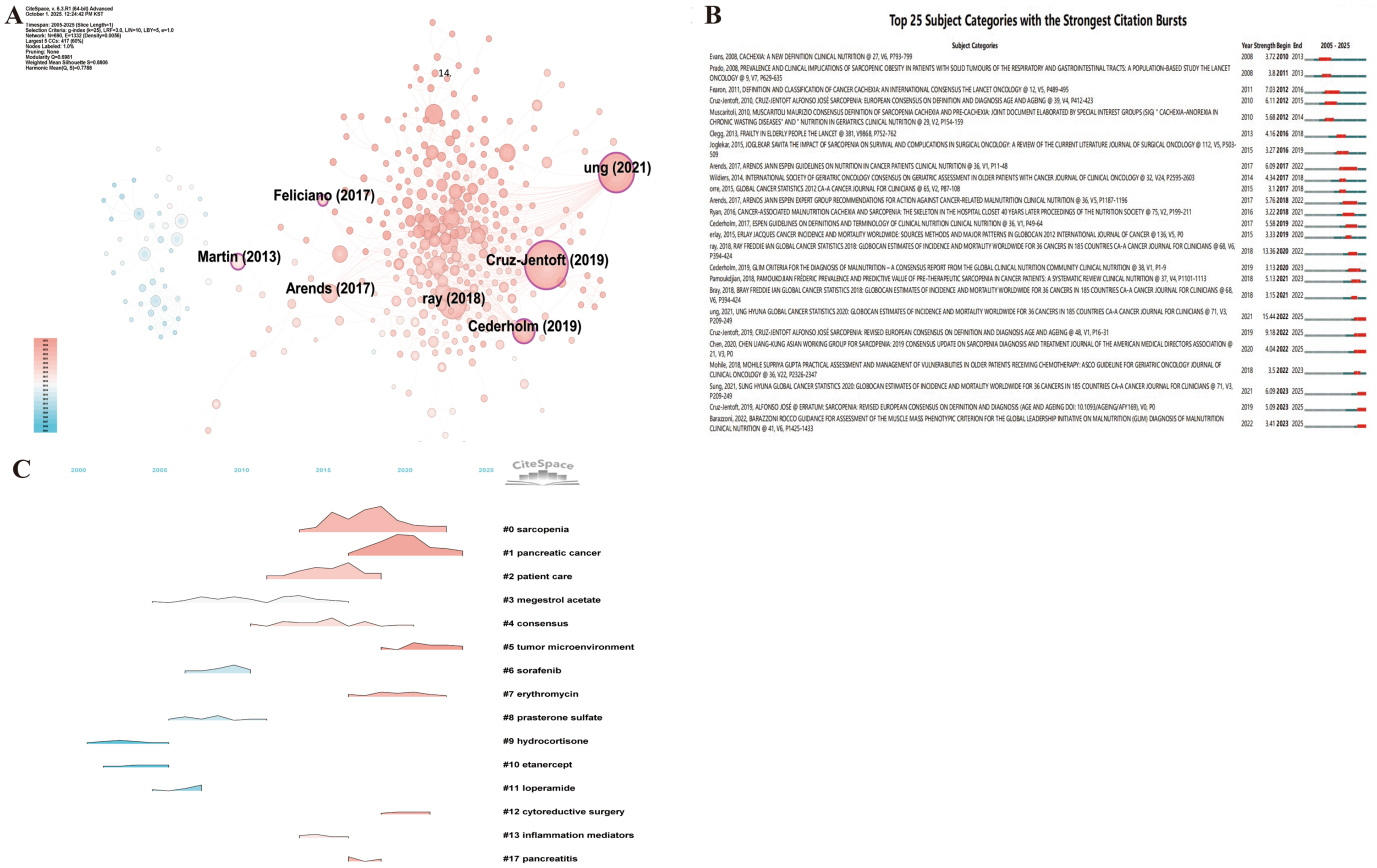
Co-citation and clustering analysis of references reveals major thematic domains and their temporal evolution. **(A)** Reference betweenness centrality network (Scopus dataset). **(B)** Top 25 references with the strongest citation bursts (Scopus dataset). **(C)** Reference clustering landscape (Scopus dataset) illustrating thematic evolution over time.

Reference analysis of the Scopus dataset further corroborated the findings from the WoSCC. Six publications exhibited high betweenness centrality (1, 41, 42, 51, 53, 56) (Figure 8A), including three consensus or guideline papers—EWGSOP2 (51), GLIM (53), and the GLOBOCAN 2020 report (1)—which were also identified as citation bursts in both databases, as well as two of the top 10 most cited papers (41, 42). This cross-database consistency reinforces the robustness of the field’s intellectual structure and highlights the pivotal role of clinical guidelines and standardized definitions in shaping contemporary research. The top 25 references with ongoing citation bursts in Scopus (Figure 8B) further confirmed these trends. Four papers remain in active burst status (1, 42, 43, 53), three of which overlap with the WoSCC results [EWGSOP2 (1), AWGS (52), and GLOBOCAN (1)]. Additionally, Barazzoni et al. (57) refined the GLIM framework by standardizing methods for assessing muscle mass phenotypes, thereby advancing the field toward quantitative, biomarker-based precision phenotyping to define malnutrition and frailty in cancer patients. Co-citation clustering of the Scopus dataset identified 18 clusters, with cluster #5 (tumor microenvironment) remaining active (Figure 8C), highlighting the current research focus on metabolic-immunologic interactions within cancer.

These findings demonstrate the evolving nature of the field’s knowledge base. The research focus has transitioned from merely identifying malnutrition to developing an integrated framework that encompasses muscle dysfunction, nutritional status, and metabolic abnormalities. The paradigm has shifted from empirical nutritional support to standardized, evidence-based assessment and intervention systems. Interdisciplinary convergence has strengthened, forming a consolidated intellectual core centered on sarcopenia, the GLIM criteria, and oncologic nutrition guidelines. This trajectory indicates that research on frailty and nutrition in cancer patients is now at a critical translational stage, progressing from conceptual refinement to precision intervention.

### 3.7 Hotspots and frontiers

**Figure 9:**
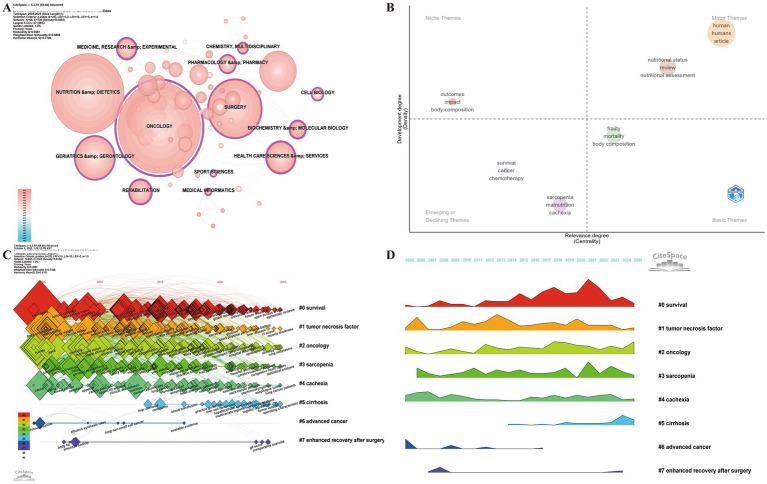
Analytical overview of research domains and keywords in the study of nutrition and frailty in cancer patients. **(A)** Betweenness centrality of disciplines (WoSCC dataset). **(B)** Thematic map showing the maturity and centrality of key topics. **(C)** Keyword timeline map illustrating the temporal evolution of research themes. **(D)** Keyword clustering landscape highlighting thematic density and interrelations.

Keyword analysis serves as key bibliometric indicator, enabling researchers to trace the evolutionary trajectory, research hotspots, and emerging trends within a scientific field (27, 58). Due to database constraints, the keyword discipline analysis in this study was performed using the WoSCC dataset. The results identified 12 disciplines with high betweenness centrality (Figure 9A), including oncology (1.08), biochemistry (0.33), health care sciences & services (0.28), rehabilitation (0.27), experimental medical research (0.26), cell biology (0.17), multidisciplinary chemistry (0.16), medical informatics (0.16), pharmacology (0.15), geriatrics (0.13), surgery (0.13), and sports sciences (0.11). These findings suggest the field has evolved from traditional oncology to a multidisciplinary landscape integrating molecular biology, clinical medicine, geriatrics, and rehabilitation sciences.

The thematic map (Figure 9B) provides a two-dimensional visualization of thematic centrality and density, illustrating the relevance and maturity of various research themes. Topics related to nutritional status and nutrition assessment are positioned in the upper right quadrant as motor themes, signifying mature and influential areas. This highlights the central role of nutritional evaluation in identifying frailty, predicting treatment tolerance, and guiding individualized interventions. Meanwhile, cancer, survival, and chemotherapy occupy the emerging themes quadrant (lower left), reflecting increasing interest in how malnutrition and frailty impact oncologic outcomes, treatment tolerance, and survival trajectories. Overall, the thematic map illustrates a transition from risk description to integrative, outcome-oriented approaches, emphasizing clinical translation and intervention development at the intersection of nutrition and frailty in oncology.

Keyword co-occurrence clustering identified eight major clusters (Figure 9C), reflecting the field's multidimensional intellectual structure, with clusters #0–#5 remaining active (Figure 9D). Cluster #0 (Survival) focuses on prognosis and patient trajectories with emerging terms such as prehabilitation, global leadership, and palliative care highlighting a trend toward optimizing pre-treatment functional status, interdisciplinary collaboration, and the integration of supportive and end-of-life care frameworks. Cluster #1 (Tumor Necrosis Factor) represents the inflammatory and metabolic mechanisms associated with frailty and nutritional decline. The appearance of CT scans and muscle thickness as new terms signals a methodological shift toward imaging-based muscle quantification, linking inflammatory biomarkers with morphological and functional indicators. Cluster #2 (Oncology) encompasses disease characteristics and healthcare outcomes, with terms like etiology, epidemiology, and hospital readmission reflecting growing interest in risk stratification and population-level analyses of frail cancer cohorts. Cluster #3 (Sarcopenia) highlights the intersection between sarcopenia and comorbidities, marked by the emergence of cerebrovascular accident and congestive heart failure, suggesting that sarcopenia is viewed as a multisystem syndrome interacting with cardiovascular and neurological diseases, exacerbating patient vulnerability. Cluster #4 (Cachexia) remains highly active, with the emergence of lung cancer patients as a new term, reflecting increasing attention to tumor-specific cachexia, particularly in thoracic malignancies with severe metabolic alterations. Cluster #5 (Cirrhosis) recently incorporated terms such as tumor volume and operating characteristic, indicating the growing use of quantitative imaging metrics to assess liver dysfunction and nutritional status, especially in hepatobiliary malignancies. Collectively, these active clusters reveal that studies on frailty and nutrition in cancer have transitioned from descriptive epidemiology toward mechanistic, precision-driven oncologic applications. The integration of inflammatory biology, imaging-based assessment, and prehabilitation strategies reflects the field’s maturation into a comprehensive framework linking biological mechanisms, functional outcomes, and multidisciplinary interventions.

**Figure 10:**
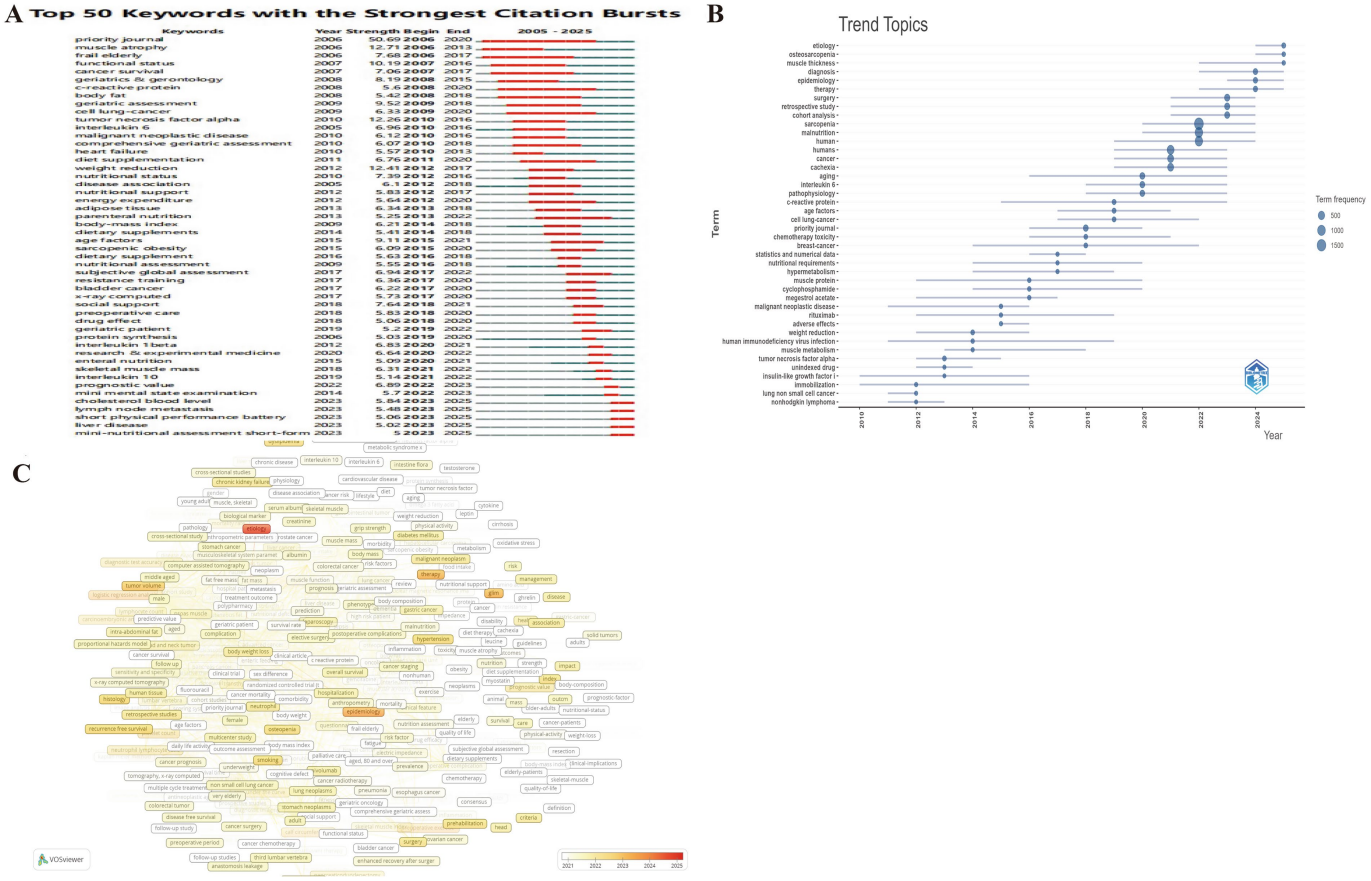
Analytical overview of research domains and keywords in the study of nutrition and frailty in cancer patients. **(A)** Top 25 keywords with the strongest citation bursts from 2005 to 2025. **(B)** Trend topic analysis showing emerging research focuses (2023–2025). **(C)** Heatmap of keywords with average appearing year from 2021 to 2025, indicating recently emerging concepts and evolving directions.

The burst keyword analysis (Figure 10A) tracks the temporal evolution of research foci, highlighting paradigm shifts over the past two decades. In the initial descriptive phase, burst terms such as muscle atrophy, frail elderly, functional status, and cancer survival represent foundational studies on the prevalence and clinical impact of frailty, malnutrition, and muscle wasting in older and cancer populations. The research focus then shifted toward comprehensive assessment and biological mechanisms. Terms like geriatric assessment, tumor necrosis factor alpha, interleukin-6, and interleukin-1β signify the incorporation of inflammatory biomarkers into frailty and cachexia research. Concurrently, diet supplementation, nutritional support, and parenteral nutrition signal growing interest in therapeutic nutrition and metabolic interventions, establishing the “frailty–inflammation–nutrition” triad as a central conceptual model. In the most recent phase, burst terms such as nutritional assessment, subjective global assessment, resistance training, x-ray computed tomography, and skeletal muscle mass highlight the adoption of standardized screening tools and imaging-based diagnostic methods to evaluate nutritional and functional decline. Terms like social support, preoperative care, and prognostic value expand the research perspective to encompass psychosocial factors and perioperative management, aligning with the reference burst analysis, which reveals that clinical guidelines and consensus statements now dominate citations. Notably, currently bursting keywords including cholesterol blood level, lymph node metastasis, short physical performance battery (SPPB), liver disease, and MNA-SF, point to a new trajectory toward biochemical, functional, and organ-specific evaluation models.

Emerging keyword analysis provides a forecast of future research frontiers. The trend topic analysis (Figure 10B) identified key priorities for 2023–2025, such as etiology, osteosarcopenia, muscle thickness, therapy, epidemiology, diagnosis, cohort analysis, retrospective study, and surgery. These terms signal a shift from theoretical modeling to mechanistic exploration, quantitative imaging, and real-world clinical validation. Analysis of keywords with an Average Appearing Year (AAY) of 2021–2025 (Figure 10C) revealed emerging terms like prevention and control, drug therapy, etiology, prognostic nutritional index (PNI), preoperative exercise, mini nutritional assessment short-form (MNA-SF), epidemiology, demographics, diagnosis, and calf circumference, highlighting an increasing focus on early prevention, precision evaluation, and individualized management. These patterns collectively reveal a unified paradigm shift toward proactive, multimodal interventions, refined diagnostics, and mechanistic and population-level investigations.

In summary, the keyword co-occurrence, clustering, burst, and trend analyses outline a rapidly evolving and increasingly translational research landscape at the intersection of frailty, nutrition, and oncology. Over the past two decades, the field has evolved from descriptive epidemiology and clinical observation to mechanism-oriented, biomarker-driven, and precision-intervention frameworks. Current research hotspots include multimodal nutrition and exercise interventions, standardized and quantitative assessment, and biomarker and mechanistic exploration. Future directions are likely to emphasize body composition analysis, omics-based biomarker discovery, and machine learning–driven prognostic modeling for achieve more individualized risk prediction.

## 4 Discussion

### 4.1 Overview of the global research landscape

Based on bibliometric and knowledge-mapping analyses, this study provides a comprehensive overview of the global research landscape on frailty and nutrition in cancer patients. It systematically summarizes the current state, research hotspots, and emerging trends, revealing a rapidly expanding and increasingly multidisciplinary field. Data from the WoSCC and Scopus databases show a substantial growth in research output over the past two decades, with a notable surge post-2020. This acceleration is likely driven by the aging global population, evolving cancer treatment paradigms, and growing recognition of the clinical importance of nutritional status and frailty in oncology. These trends align with the development of standardized diagnostic frameworks and evidence-based clinical guidelines. On a macro scale, scientific output remains concentrated in developed regions such as the United States, Europe, and East Asia, where academic institutions and clinical research centers are deeply engaged in international collaborations. However, the relatively low rate of international cooperation suggests the need for increased global collaboration. Leading journals such as Nutrients, Clinical Nutrition, and the Journal of Cachexia, Sarcopenia and Muscle serve as key platforms, highlighting the interdisciplinary integration of nutritional science, gerontology, and oncology. Researchers aiming to track emerging topics or forge collaborations can benefit from monitoring key contributors, institutions, and journals within these networks.

Reference analysis provides valuable insights into the historical and structural foundations of the field, offering an evidence base for future directions and strategic priorities. The 10 most-cited publications (41–50), spanning 2008 to 2023, collectively trace the conceptual evolution of the field—from early clinical recognition and frailty screening to mechanistic exploration and international standardization. These studies primarily focus on conceptualization, biological mechanisms, standardization, and clinical application, emphasizing that nutritional assessment and targeted interventions are key strategies for mitigating frailty, enhancing resilience, and improving survival and quality of life in cancer patients. Reference clustering further highlights a shift from a narrow focus on malnutrition identification to a more integrated framework that encompasses muscle dysfunction, nutritional status, and metabolic dysregulation. Notably, cluster #0 (GLIM) and cluster #5 (head and neck Cancer) have expanded rapidly in recent years, positioning them as current research hotspots.

### 4.2 Hotspots and frontiers

The core value of bibliometric analysis lies in its ability to identify research hotspots and developmental trajectories. Keyword co-occurrence analysis revealed that oncology, biochemistry, healthcare sciences, rehabilitation, experimental medicine, and cell biology form the scientific foundation of frailty and nutrition research in oncology. Over the past two decades, the field has evolved from descriptive epidemiology to mechanistic exploration and, more recently, to translational and precision-driven paradigms. This shift reflects an increasing recognition of frailty as a determinant of cancer prognosis, alongside the integration of nutritional science, geriatrics, and oncology into a multidisciplinary framework. Early research focused on describing the prevalence and clinical consequences of malnutrition, sarcopenia, and cachexia, while later studies emphasized inflammatory and metabolic mechanisms. Recent efforts have prioritized standardized diagnostic criteria and multimodal management strategies, combining exercise, nutrition, pharmacological, and psychosocial interventions. The inclusion of imaging-based body composition assessments highlights the trend toward quantitative and personalized evaluation.

Emerging keywords such as etiology, epidemiology, liver disease, lymph node metastasis, and cholesterol blood level represent the forefront of precision assessment and prognostic modeling in the field. Additionally, the growing emphasis on prevention and control, preoperative exercise, and drug therapy signals a proactive shift toward resilience-building in cancer care. In summary, research on frailty and nutrition in oncology has steadily progressed from conceptual understanding to clinical application. The emerging knowledge framework, anchored in standardized diagnostics, mechanistic insights, and multimodal interventions, marks a pivotal step toward personalized and preventive oncology, aligning with global health priorities in aging and cancer survivorship.

#### 4.2.1 The role of multimodal nutrition and exercise interventions in improving frailty and quality of life in patients with cancer

The first research hotspot identified in this study highlights a paradigm shift in cancer patient management—from isolated nutritional support to comprehensive, integrative intervention frameworks. This trend is reflected in emerging keywords such as prehabilitation, preoperative exercise, prevention and control, therapy, and drug therapy, alongside the rapid growth of Cluster #0 (GLIM) in reference clustering analysis. These findings collectively highlight the growing recognition that frailty and malnutrition are complex, multifactorial syndromes requiring synergistic interventions that address both physiological and psychosocial vulnerabilities.

Multimodal interventions, which combine nutritional therapy with structured exercise, have emerged as one of the most promising strategies for alleviating frailty and improving clinical outcomes in oncology. The core principle is to maximize the synergistic effects between nutritional supplementation and physical rehabilitation. Numerous randomized controlled trials (RCTs) and cohort studies have demonstrated that isolated nutritional supplementation has limited efficacy in reversing cancer cachexia (with an objective response rate < 30%) (59). In contrast, integrated programs that combine individualized dietary counseling, high-protein supplementation, and structured exercise (including resistance, aerobic, and balance training) can increase muscle mass by 50–60% (34, 36). These combined approaches significantly enhance physical performance, immune function, sleep quality, and psychological well-being, while reducing inflammation, postoperative complications, fatigue, and mortality, ultimately improving patients’ overall quality of life (33, 59, 60). The synergistic benefits are thought to result from exercise-induced anabolic hormone secretion, reduced chronic systemic inflammation, and improved metabolic utilization of nutritional substrates (61).

Notably, multimodal interventions often include psychological support, symptom management, and pharmacologic therapy, embodying a holistic, patient-centered care model. This aligns with the contemporary concept of prehabilitation, which focuses on initiating interventions before oncological treatments (such as surgery or chemotherapy) to optimize baseline functional capacity, reduce postoperative complications, and shorten hospital stays (40, 60, 61). For instance, a multicenter Chinese study involving 184 elderly gastric cancer patients demonstrated that a two-week preoperative multimodal prehabilitation program—comprising physical and respiratory exercises, nutritional optimization, and psychosocial support—significantly improved functional performance, reduced surgical stress and inflammatory complications, and may have modulated the tumor microenvironment, leading to better short- and long-term outcomes (61).

In summary, multimodal intervention represents a cornerstone of precision supportive oncology, integrating rehabilitation science, nutrition, and psychosocial care to enhance recovery and survival. However, several challenges remain. First, standardization is lacking—studies vary significantly in nutritional formulations, exercise type, frequency, and intensity, hindering comparability and generalizability. Second, personalization remains insufficient; few studies consider patient-specific factors such as cancer type, disease stage, treatment modality, and baseline functional capacity when tailoring interventions. Third, adherence is a major barrier—data show that over 40% of patients discontinue interventions within 6 months (61, 62). To address these limitations, future research should focus on developing AI-assisted and wearable technology–based intelligent intervention systems, designing behavioral strategies to enhance adherence, and conducting large-scale, multicenter RCTs to validate the long-term sustainability and cost-effectiveness of multimodal approaches. Nevertheless, several obstacles currently limit the integration of AI and wearable technologies into routine clinical practice, including data privacy concerns, lack of standardized platforms, limited interoperability with hospital information systems, and the need for clinician training and workflow adaptation. Ultimately, the integration of data-driven personalization and technological innovation will enable a new generation of adaptive, precision-guided frailty interventions in oncology.

#### 4.2.2 Standardization and optimization of frailty and nutritional assessment in patients with cancer

The second major research hotspot centers on the standardization of frailty, sarcopenia, and malnutrition assessments, a focus strongly supported by citation burst and thematic mapping analyses. This study identifies the EWGSOP2, AWGS, GLIM, and ESPEN consensus frameworks as the most frequently cited documents, marking a methodological turning point in the field. These frameworks established internationally recognized diagnostic criteria that unify previously fragmented definitions, enabling cross-study comparability. Concurrently, keywords such as muscle thickness, MNA-SF, PNI, body composition, SPPB, and calf circumference have experienced citation bursts, while terms like nutritional status and nutrition assessment are positioned in the upper-right quadrant of the thematic map, representing well-established and influential motor themes driving both clinical implementation and research harmonization.

Standardized tools facilitate systematic screening of high-risk populations, quantitative monitoring of disease trajectories, and evaluation of intervention efficacy. Several screening and diagnostic instruments are currently in use to assess frailty and nutritional status in cancer patients, including the Fried Frailty Phenotype, Clinical Frailty Scale (CFS), Mini Nutritional Assessment (MNA), and PG-SGA (5, 32, 35, 38, 39). Structured malnutrition assessment tools have become essential components of supportive oncology, enabling early identification of patients at nutritional risk and supporting individualized intervention strategies. Recent studies have emphasized the clinical value of standardized nutritional assessment instruments for improving prognostic stratification and guiding treatment decision-making in cancer populations (14–16). Numerous studies have demonstrated strong associations between these assessments and clinical outcomes, such as hospital stay duration, postoperative complications, chemotherapy tolerance, and survival (5, 32, 35, 38, 39). However, the heterogeneity in sensitivity and specificity across these tools contributes to inconsistent prevalence estimates and outcome correlations. Most frailty instruments originate from geriatric medicine, capturing core phenotypic dimensions such as unintentional weight loss, reduced grip strength, slower gait speed, fatigue, and lower activity levels. These instruments, however, are less sensitive to cancer-specific manifestations, including chemotherapy-related cognitive decline (“chemo brain”), immunotherapy-induced fatigue, and gastrointestinal toxicity from targeted therapies. Knowledge mapping indicates that researchers are developing oncology-specific frailty assessment tools, though these tools require further clinical validation regarding predictive accuracy, cutoff determination, and longitudinal performance. Similarly, while the GLIM criteria aim to standardize malnutrition diagnosis globally, their applicability and prognostic validity in oncology populations remain to be confirmed (39).

Integrating frailty and nutritional assessments into routine clinical workflows for early screening and dynamic monitoring remains a practical challenge. Recent advances in automated assessment technologies—leveraging electronic health records, machine learning algorithms, and wearable devices—offer promising solutions. Predictive models that combine functional parameters (e.g., handgrip strength, gait speed) with body composition metrics derived from BIA, DXA, or CT scans can identify high-risk patients and guide individualized care plans (10). Additionally, wearable activity monitors can track physical activity and sleep quality in real-time, facilitating continuous, objective assessment (32, 63).

Overall, frailty and nutritional assessment in oncology is evolving toward multi-cancer, multidimensional, and digitized frameworks. However, current tools still lack validation in specific subgroups and often suffer from complexity, time demands, and subjective bias. Future studies should focus on multicenter prospective trials to validate predictive thresholds across various cancer types and treatment stages, develop simplified, sensitive, and cancer-specific frailty screening tools for rapid outpatient use, and leverage digital health technologies for automated and remote evaluation. These advancements will lay the foundation for personalized, dynamic, and precision-guided frailty management in cancer care.

#### 4.2.3 Biomarkers and mechanistic insights into malnutrition and frailty in cancer

The third emerging frontier focuses on elucidating the biochemical and molecular biomarkers underlying the pathophysiological interplay between malnutrition and frailty in cancer patients, aiming to identify objective indicators for early diagnosis, risk stratification, and treatment monitoring. Keyword burst analysis revealed ongoing citation growth for terms such as cholesterol blood level, lymph node metastasis, PNI, diagnosis, muscle thickness, etiology, and disease association, while reference clustering identified Cluster #5 (tumor microenvironment) as an active and expanding research area, highlighting the field’s increasing focus on inflammatory and metabolic pathways.

The discovery and validation of biomarkers are critical steps toward precision oncology. Current biomarker research spans multiple biological domains: inflammatory markers, including C-reactive protein (CRP), interleukin-6 (IL-6), and tumor necrosis factor-*α* (TNF-α), reflect tumor-associated chronic inflammation, which is closely linked to muscle catabolism and metabolic dysregulation (64); muscle-related markers, such as creatine kinase and myostatin, directly capture changes in muscle mass and quality; and metabolic markers, including albumin, prealbumin, and transferrin, reflect protein-energy status and systemic metabolism (11, 31, 63, 65). Emerging molecular markers, such as circulating microRNAs and exosomes, offer deeper insights into regulatory mechanisms (66). Evidence increasingly suggests that single or composite biomarker panels can effectively predict frailty, malnutrition, and clinical outcomes in cancer patients (30, 67).

Recent studies have integrated these biomarkers with imaging-derived indices—such as skeletal muscle index and muscle thickness, and functional measures (e.g., SPPB, handgrip strength) to develop multidimensional predictive models (10). Composite inflammatory-nutritional scores, including the Glasgow Prognostic Score (GPS), modified GPS (mGPS), and PNI have demonstrated robust prognostic value across multiple tumor types (37, 68, 69). Notably, investigations into the inflammation–nutrition–frailty axis have deepened our understanding of frailty pathogenesis. Tumor-induced chronic inflammation not only promotes muscle proteolysis but also drives anorexia and metabolic shifts, creating a vicious cycle of malnutrition and physical decline. These insights have spurred interest in targeting inflammatory pathways as therapeutic strategies, using nonsteroidal anti-inflammatory drugs, selective COX-2 inhibitors, or anti-IL-6 monoclonal antibodies, to prevent or mitigate frailty progression (70).

In summary, biomarkers hold significant promise for improving the prediction and management of frailty and malnutrition in cancer patients. However, current biomarker research is still in the early developmental stage. Most markers lack specificity, are influenced by multiple confounding factors, and suffer from inconsistent methodologies and threshold standards, limiting comparability across studies. Furthermore, the translation from discovery to clinical application remains unclear due to a lack of prospective validation studies. Future research should focus on large-scale prospective cohorts to evaluate the diagnostic accuracy, prognostic utility, and clinical feasibility of candidate biomarkers. Additionally, exploring the application of biomarkers to guide individualized interventions and monitor therapeutic responses, as well as elucidating the causal mechanisms linking biomarkers to disease pathways, will be essential for identifying novel therapeutic targets for early detection and tailored nutritional strategies. Such translational efforts will contribute to the development of precision oncology models that position metabolic resilience as a therapeutic target, bridging molecular insights with clinical implementation in cancer-related frailty management.

#### 4.2.4 Translational implications: linking bibliometric shifts to clinical and policy changes

This bibliometric study not only maps the scientific landscape and knowledge structure of frailty and nutrition research in cancer but also provides comprehensive evidence to guide clinical optimization, policy development, and strategic research planning.

From a clinical perspective, the findings highlight the urgent need for standardized screening and assessment frameworks across the cancer care continuum. Implementing early risk stratification and comprehensive evaluation protocols will improve both accuracy and feasibility in routine practice. Additionally, the increasing focus on multimodal and multidisciplinary management highlights the need for coordinated care teams, including oncology, nutrition, rehabilitation, psychology, and pharmacy specialists, to create individualized intervention plans. The emergence of prehabilitation as a research frontier signals a shift from reactive to proactive care, emphasizing early functional optimization before surgery or chemotherapy. Concurrently, the use of validated biomarkers will further enable personalized risk stratification and precision interventions in clinical oncology.

From a policy perspective, the results highlight the importance of integrating frailty and nutritional management into national oncology care frameworks and clinical pathways. Policymakers should allocate dedicated research funding to support translational and implementation studies, including large-scale clinical trials and real-world evaluations, while fostering international collaboration and knowledge exchange. Ultimately, strengthening the integration of research, clinical practice, and health policy will advance patient-centered care, improving survival, functionality, and quality of life for frail cancer patients.

#### 4.2.5 Limitations

This bibliometric analysis provides a comprehensive overview of global research trends, collaborative networks, and thematic evolution in cancer-related frailty and nutrition. However, several limitations should be acknowledged. First, this study did not stratify data by cancer type, stage, or treatment modality—factors that may distinctly influence the trajectory of frailty and nutritional decline. Future studies should conduct more granular analyses across cancer subtypes, treatment phases (surgery, chemotherapy, radiotherapy), and age groups. Second, due to current database constraints, certain analyses could not be fully harmonized across data sources. The development of more integrated, cross-database analytic tools will be crucial for future bibliometric and meta-scientific research.

## 5 Conclusion

This bibliometric study offers a systematic and comprehensive overview of global research on frailty and nutrition in cancer patients over the past two decades. By integrating data from both WoSCC and the Scopus databases, the study maps the scientific development, knowledge structure, and emerging frontiers in the field. The findings reveal a clear evolution from descriptive studies on malnutrition and frailty to mechanistic exploration, and more recently, to standardized, evidence-based, and precision-oriented clinical models. The frequent citation of four major international guidelines reflects a growing global consensus toward standardized frameworks for the assessment and management of sarcopenia, malnutrition, and frailty. Thematic and keyword analyses show that nutritional status and nutritional assessment are research hotspots in the field, while emerging studies increasingly emphasize prehabilitation, multimodal therapy, and functional recovery. Recent burst keywords, such as osteosarcopenia, muscle thickness, etiology, preoperative exercise, and MNA-SF indicate a shift toward precision assessment, early intervention, and personalized rehabilitation. These trends signal the onset of a new era of translational integration across oncology, geriatrics, and nutrition science.

Clinically, the findings highlight the importance of early frailty detection, routine nutritional screening, and the incorporation of imaging-based and functional metrics into comprehensive cancer management. At the policy level, the study advocates for embedding frailty and nutrition management within national cancer strategies. In conclusion, the intersection of frailty and nutrition research represents a critical frontier in geriatric oncology. The field is progressing toward precision, prevention, and patient-centered paradigms, integrating biological mechanisms, functional capacity, and nutritional interventions to optimize quality of life and survival outcomes.

## Data Availability

The raw data supporting the conclusions of this article will be made available by the authors, without undue reservation.

## References

[1] Sung H Ferlay J Siegel RL Laversanne M Soerjomataram I Jemal A . Global cancer statistics 2020: globocan estimates of incidence and mortality worldwide for 36 cancers in 185 countries. CA Cancer J Clin. (2021) 71:209–49. doi: 10.3322/caac.21660 33538338

[2] Hanssen D Champion N Ngo J Palfi S Whiting J Sun W . Frailty and malnutrition in surgical outcomes of elderly breast cancer patients. J Surg Oncol. (2025) 131:349–55. doi: 10.1002/jso.27940 39387508

[3] Humphry N Wilson T Bye K Draper J Hewitt J. 2324 feasibility of screening for frailty, sarcopenia and nutritional status in elective surgery for colorectal cancer. Age Ageing. (2024) 53:iii25. doi: 10.1093/ageing/afae139.093

[4] Dlima SD Hall A Aminu AQ Akpan A Todd C Vardy E. Frailty: a global health challenge in need of local action. BMJ Glob Health. (2024) 9:1–11. doi: 10.1136/bmjgh-2024-015173, 39122463 PMC11331888

[5] Dogan O Sahinli H Yazilitas D. Assessment of malnutrition in Cancer patients: a geriatric approach with the Mini nutritional assessment. Front Nutr. (2025) 12:1590137. doi: 10.3389/fnut.2025.1590137, 40416373 PMC12101082

[6] Dewansingh P Bras L Ter Beek L Krijnen WP Roodenburg JLN van der Schans CP . Malnutrition risk and frailty in head and neck cancer patients: coexistent but distinct conditions. Eur Arch Otorrinolaringol. (2023) 280:1893–902. doi: 10.1007/s00405-022-07728-6, 36484854 PMC9988738

[7] Neto L Enriquez-Martinez OG Grippa WR Marcarini JAC Santos TB Mawandji NBS . Nutritional status of patients with neoplasms undergoing ambulatory chemotherapy and associated factors. Nutrients. (2025) 17:168. doi: 10.3390/nu17010168 39796602 PMC11723305

[8] Dogan Akagunduz D Sahin H Akagunduz B. Malnutrition and related factors in older patients with gastrointestinal Cancer receiving chemotherapy. Cureus. (2024) 16:e58252. doi: 10.7759/cureus.58252, 38745807 PMC11093618

[9] Akagunduz B Ergun Y Dog An Akagunduz DD Akbas N Akagunduz D Karaog Lu A . Blood-based biomarkers of frailty in older patients with cancer. Curr Opin Support Palliat Care. (2025) 19:25–32. doi: 10.1097/SPC.0000000000000747 39888831

[10] Kaur J Wookey V Meeker CR Connors MM Handorf EA Tejani MA . Assessment of nutritional status in elderly patients with gastroesophageal cancer: a post hoc analysis. J Clin Oncol. (2025) 43:389. doi: 10.1200/JCO.2025.43.4_suppl.389

[11] Chauhan S Langstraat CL Fought AJ McGree ME Cliby WA Kumar A. Relationship between frailty and nutrition: refining predictors of mortality after primary cytoreductive surgery for ovarian cancer. Gynecol Oncol. (2024) 180:126–31. doi: 10.1016/j.ygyno.2023.11.031 38091771

[12] Dos Reis PF Martucci RB. Factors influencing health-related quality of life in patients with bladder or kidney cancer: a prospective cohort study of the impact of nutritional status and frailty phenotype. J Cancer Surviv. (2025) 19:743–53. doi: 10.1007/s11764-024-01637-9 38954250

[13] Zhang M Wang J Li X Zhang L Zhang Y Wen Z . Association between dietary supplement use and mortality in Cancer survivors with different body mass index and frailty status: a cohort study. Front Nutr. (2024) 11:1395362. doi: 10.3389/fnut.2024.1395362, 38751742 PMC11094810

[14] de la Torre-Montero JC Serra-Lopez J Alvarez-Garcia R. Nutritional status in locally advanced or metastatic solid cancer patients treated with chemotherapy, radiotherapy, and immunotherapy in Spanish outpatient oncology units. Semin Oncol Nurs. (2025) 41:151801. doi: 10.1016/j.soncn.2024.151801 39794238

[15] Rasschaert M Vandecandelaere P Marechal S D'Hondt R Vulsteke C Mailleux M . Malnutrition prevalence in cancer patients in Belgium: the Oncocare study. Support Care Cancer. (2024) 32:135. doi: 10.1007/s00520-024-08324-6, 38280135 PMC10821821

[16] Zhang J Quan Y Wang X Wei X Shen X Li X . Global epidemiological characteristics of malnutrition in Cancer patients: a comprehensive Meta-analysis and systematic review. BMC Cancer. (2025) 25:1191. doi: 10.1186/s12885-025-14558-2, 40684092 PMC12275410

[17] Ripa C Olariaga O Vallinas S Sirvent M Prado E Romero R . Onconutridos study: prevalence of disease-related malnutrition according to Glim criteria in cancer patients in Oncohaematology day hospitals. Clin Nutr ESPEN. (2025) 70:666–75. doi: 10.1016/j.clnesp.2025.11.008 41238020

[18] Wada Y Nishi M Yoshikawa K Takasu C Tokunaga T Nakao T . Preoperative nutrition and exercise intervention in frailty patients with gastric cancer undergoing gastrectomy. Int J Clin Oncol. (2022) 27:1421–7. doi: 10.1007/s10147-022-02202-z 35771395

[19] Vella R Pizzocaro E Bannone E Gualtieri P Frank G Giardino A . Nutritional intervention for the elderly during chemotherapy: a systematic review. Cancers (Basel). (2024) 16:2809. doi: 10.3390/cancers16162809, 39199582 PMC11352472

[20] Donthu N Kumar S Mukherjee D Pandey N Lim WM. How to conduct a bibliometric analysis: an overview and guidelines. J Bus Res. (2021) 133:285–96. doi: 10.1016/j.jbusres.2021.04.070

[21] Zhang F Xiong Y Meng X Xu H Zhang Q. Bibliometric analysis of comprehensive geriatric assessment from 2004 to 2023. J Multidiscip Healthc. (2024) 17:5901–15. doi: 10.2147/jmdh.S488030, 39678715 PMC11645894

[22] Liu R Wu M Cheng X Zhou C Cheng C Feng L . Global trends in sarcopenia and cancer over the past 10 years: a bibliometric analysis. Discov Oncol. (2025) 16:1358. doi: 10.1007/s12672-025-03185-9, 40676319 PMC12271044

[23] Chen Y Chen X Zhong L Lu H Zhang H Jiang M. Bibliometric analysis of research trends in the relationship between frailty and neoplasms over the past decade. Support Care Cancer. (2024) 32:536. doi: 10.1007/s00520-024-08744-4, 39042180 PMC11266222

[24] Shi L Tian X Bongsook Y Chen J. Predicting the unseen: nutritional interventions as a key to combat frailty. Front Nutr. (2025) 12:1575922. doi: 10.3389/fnut.2025.1575922, 40704314 PMC12283294

[25] Aria M Cuccurullo C. Bibliometrix: an R-tool for comprehensive science mapping analysis. J Inf Secur. (2017) 11:959–75. doi: 10.1016/j.joi.2017.08.007

[26] Chen C Ibekwe-SanJuan F Hou J. The structure and dynamics of cocitation clusters: a multiple-perspective cocitation analysis. J Am Soc Inf Sci Technol. (2010) 61:1386–409. doi: 10.1002/asi.21309

[27] Chen C Song M. Visualizing a field of research: a methodology of systematic Scientometric reviews. PLoS One. (2019) 14:e0223994. doi: 10.1371/journal.pone.0223994, 31671124 PMC6822756

[28] Chen CM. Citespace II: detecting and visualizing emerging trends and transient patterns in scientific literature. J Am Soc Inf Sci Technol. (2006) 57:359–77. doi: 10.1002/asi.20317

[29] van Eck NJ Waltman L. Software survey: Vosviewer, a computer program for bibliometric mapping. Scientometrics. (2010) 84:523–38. doi: 10.1007/s11192-009-0146-3, 20585380 PMC2883932

[30] Kapala A Rozycka K Grochowska E Gazi A Motacka E Folwarski M. Cancer, malnutrition and inflammatory biomarkers. Why do some Cancer patients lose more weight than others? Contemp Oncol (Pozn). (2025) 29:45–54. doi: 10.5114/wo.2025.147939, 40330451 PMC12051885

[31] Enkobahry A Sime T Kene K Mateos T Dilnesa S Zawdie B. Blood biomarkers as potential malnutrition screening alternatives among adult patients with Cancer on treatment in oncology unit of Jimma tertiary hospital: a cross-sectional analysis. BMC Nutr. (2023) 9:38. doi: 10.1186/s40795-023-00694-0, 36869395 PMC9982783

[32] Patel I Winer A. Assessing frailty in gastrointestinal cancer: two diseases in one? Curr Oncol Rep. (2024) 26:90–102. doi: 10.1007/s11912-023-01483-5 38180691

[33] Fernandez-Rodriguez EJ Sanchez-Gomez C Mendez-Sanchez R Recio-Rodriguez JI Puente-Gonzalez AS Gonzalez-Sanchez J . Multimodal physical exercise and functional rehabilitation program in oncological patients with cancer-related fatigue-a randomized clinical trial. Int J Environ Res Public Health. (2023) 20:2809. doi: 10.3390/ijerph20064938, 36981846 PMC10049732

[34] Du L Liu X Zhu Q Zhu K Li P. Sarcopenia as a prognostic factor and multimodal interventions in breast Cancer. Int J Gen Med. (2024) 17:6605–16. doi: 10.2147/IJGM.S497897, 39764482 PMC11701243

[35] Chonmaitree P Sudcharoen A Poonyam P Laoarphasuwong N Khuancharee K Thititagul O. Comparison of patient-generated subjective global assessment (Pg-Sga) and mini nutritional assessment (Mna) for nutritional assessment in hepatocellular carcinoma patients. Support Care Cancer. (2025) 33:116. doi: 10.1007/s00520-025-09176-4 39836246

[36] Maurer T Belau MH Zyriax BC Welsch G Jagemann B Chang-Claude J . Study protocol of an exercise and nutrition intervention for ovarian cancer patients during and after first-line chemotherapy (Benita) - a randomized controlled trial. BMC Cancer. (2024) 24:1379. doi: 10.1186/s12885-024-13102-y, 39528997 PMC11552146

[37] Leo S Marinelli F Zurlo IV Guarini V Accettura C Falco A . Bioimpedentiometry parameters used as indicators of frailty and malnutrition: association between G8 score and phase angle (Pha) in elderly cancer patients. Aging Clin Exp Res. (2023) 35:2219–25. doi: 10.1007/s40520-023-02512-w 37626230

[38] Arimizu K Kusumoto Y Okumura Y Komoda M Esaki T Nishijima TF. Frailty assessed by a 10-item index from comprehensive geriatric assessment (fi-Cga-10) versus oncologist-assessed performance status (Ps) in older adults with gastrointestinal (Gi) cancer. J Clin Oncol. (2025) 43:821. doi: 10.1200/JCO.2025.43.4_suppl.82139561316

[39] Zhang X Tang M Zhang Q Zhang KP Guo ZQ Xu HX . The Glim criteria as an effective tool for nutrition assessment and survival prediction in older adult cancer patients. Clin Nutr. (2021) 40:1224–32. doi: 10.1016/j.clnu.2020.08.004 32826109

[40] Kasahara R Morishita S Fujita T Jinbo R Kubota J Takano A . Effect of combined exercise and nutrition interventions during inpatient chemotherapy in acute leukemia and malignant lymphoma patients: a randomized controlled trial. Nutr Cancer. (2025) 77:115–23. doi: 10.1080/01635581.2024.2406043 39300733

[41] Martin L Birdsell L Macdonald N Reiman T Clandinin MT McCargar LJ . Cancer cachexia in the age of obesity: skeletal muscle depletion is a powerful prognostic factor, independent of body mass index. J Clin Oncol. (2013) 31:1539–47. doi: 10.1200/JCO.2012.45.2722 23530101

[42] Arends J Bachmann P Baracos V Barthelemy N Bertz H Bozzetti F . Espen guidelines on nutrition in cancer patients. Clin Nutr. (2017) 36:11–48. doi: 10.1016/j.clnu.2016.07.015 27637832

[43] Houston DK Nicklas BJ Ding J Harris TB Tylavsky FA Newman AB . Dietary protein intake is associated with lean mass change in older, community-dwelling adults: the health, aging, and body composition (health Abc) study. Am J Clin Nutr. (2008) 87:150–5. doi: 10.1093/ajcn/87.1.150 18175749

[44] Hamaker ME Jonker JM de Rooij SE Vos AG Smorenburg CH van Munster BC. Frailty screening methods for predicting outcome of a comprehensive geriatric assessment in elderly patients with cancer: a systematic review. Lancet Oncol. (2012) 13:e437–44. doi: 10.1016/S1470-2045(12)70259-0 23026829

[45] Yuan S Larsson SC. Epidemiology of sarcopenia: prevalence, risk factors, and consequences. Metabolism. (2023) 144:155533. doi: 10.1016/j.metabol.2023.155533 36907247

[46] Bellera CA Rainfray M Mathoulin-Pelissier S Mertens C Delva F Fonck M . Screening older cancer patients: first evaluation of the G-8 geriatric screening tool. Ann Oncol. (2012) 23:2166–72. doi: 10.1093/annonc/mdr587 22250183

[47] Arends J Bachmann P Baracos V Bartz H Bozzetti F Calder PC . Espen expert group recommendations for action against cancer-related malnutrition. Clin Nutr. (2017) 36:1187–96. doi: 10.1016/j.clnu.2017.06.017 28689670

[48] Cohen S Nathan JA Goldberg AL. Muscle wasting in disease: molecular mechanisms and promising therapies. Nat Rev Drug Discov. (2015) 14:58–74. doi: 10.1038/nrd4467 25549588

[49] Michaud M Balardy L Moulis G Gaudin C Peyrot C Vellas B . Proinflammatory cytokines, aging, and age-related diseases. J Am Med Dir Assoc. (2013) 14:877–82. doi: 10.1016/j.jamda.2013.05.009 23792036

[50] Muscaritoli M Anker SD Argiles J Aversa Z Bauer JM Biolo G . Consensus definition of sarcopenia, cachexia and pre-cachexia: joint document elaborated by special interest groups (sig) "Cachexia-anorexia in chronic wasting diseases" and "nutrition in geriatrics". Clin Nutr. (2010) 29:154–9. doi: 10.1016/j.clnu.2009.12.004 20060626

[51] Cruz-Jentoft AJ Bahat G Bauer J Boirie Y Bruyere O Cederholm T . Sarcopenia: revised European consensus on definition and diagnosis. Age Ageing. (2019) 48:16–31. doi: 10.1093/ageing/afy169, 30312372 PMC6322506

[52] Chen LK Woo J Assantachai P Auyeung TW Chou MY Iijima K . Asian working group for sarcopenia: 2019 consensus update on sarcopenia diagnosis and treatment. J Am Med Dir Assoc. (2020) 21:300–7 e2. doi: 10.1016/j.jamda.2019.12.012 32033882

[53] Cederholm T Jensen GL Correia M Gonzalez MC Fukushima R Higashiguchi T . Glim criteria for the diagnosis of malnutrition - a consensus report from the global clinical nutrition community. J Cachexia Sarcopenia Muscle. (2019) 10:207–17. doi: 10.1002/jcsm.12383, 30920778 PMC6438340

[54] Muscaritoli M Arends J Bachmann P Baracos V Barthelemy N Bertz H . Espen practical guideline: clinical nutrition in cancer. Clin Nutr. (2021) 40:2898–913. doi: 10.1016/j.clnu.2021.02.005 33946039

[55] Bossi P Delrio P Mascheroni A Zanetti M. The Spectrum of malnutrition/Cachexia/sarcopenia in oncology according to different Cancer types and settings: a narrative review. Nutrients. (2021) 13:1980. doi: 10.3390/nu13061980, 34207529 PMC8226689

[56] Feliciano EMC Kroenke CH Meyerhardt JA Prado CM Bradshaw PT Kwan ML . Association of systemic inflammation and sarcopenia with survival in nonmetastatic colorectal cancer: results from the C scans study. JAMA Oncol. (2017) 3:e172319. doi: 10.1001/jamaoncol.2017.2319, 28796857 PMC5824285

[57] Barazzoni R Jensen GL Correia M Gonzalez MC Higashiguchi T Shi HP . Guidance for assessment of the muscle mass phenotypic criterion for the global leadership initiative on malnutrition (Glim) diagnosis of malnutrition. Clin Nutr. (2022) 41:1425–33. doi: 10.1016/j.clnu.2022.02.001 35450768

[58] Ellegaard O Wallin JA. The bibliometric analysis of scholarly production: how great is the impact? Scientometrics. (2015) 105:1809–31. doi: 10.1007/s11192-015-1645-z, 26594073 PMC4643120

[59] Mikkelsen MK Lund CM Vinther A Tolver A Johansen JS Chen I . Effects of a 12-week multimodal exercise intervention among older patients with advanced Cancer: results from a randomized controlled trial. Oncologist. (2022) 27:67–78. doi: 10.1002/onco.13970, 34498352 PMC8842365

[60] Wu J Zhang C Jing Z Wu X. Randomized controlled trial investigating the effect of a Baduanjin exercise plus nutrition programme on cancer-related fatigue in elderly lung cancer patients receiving chemotherapy. Exp Gerontol. (2025) 206:112763. doi: 10.1016/j.exger.2025.112763 40305876

[61] Sun Y Tian Y Cao S Li L Yu W Ding Y . Multimodal prehabilitation to improve the clinical outcomes of frail elderly patients with gastric Cancer: a study protocol for a multicentre randomised controlled trial (Gissg(+)2201). BMJ Open. (2023) 13:e071714. doi: 10.1136/bmjopen-2023-071714, 37816552 PMC10565164

[62] Yang L Wen-bo W Xiong Y Cao D Dai J Xu H . Mobile-based multimodal rehabilitation including exercise, nutritional, and psychological interventions in patients with abdominal cancer receiving concurrent chemoradiotherapy: prospective, multicenter, randomized trial. J Clin Oncol. (2023) 41:e24138. doi: 10.1200/JCO.2023.41.16_suppl.e24138

[63] Wang F Zhen HN Wang HP Yu K. Measurement of sarcopenia in lung Cancer inpatients and its association with frailty, nutritional risk, and malnutrition. Front Nutr. (2023) 10:1143213. doi: 10.3389/fnut.2023.1143213, 37139454 PMC10149728

[64] Xia SF Liu Y Chen Y Li ZY Cheng L He JY . Association between dietary inflammatory potential and frailty is mediated by inflammation among patients with colorectal cancer: a cross-sectional study. Nutr Res. (2024) 125:79–90. doi: 10.1016/j.nutres.2024.03.001 38552503

[65] Zwart AT Kok LMC de Vries J van Kester MS Dierckx R de Bock GH . Radiologically defined sarcopenia as a biomarker for frailty and malnutrition in head and neck skin cancer patients. J Clin Med. (2023) 12:3445. doi: 10.3390/jcm12103445 37240550 PMC10218972

[66] Dong J Yu J Li Z Gao S Wang H Yang S . Serum insulin-like growth factor binding protein 2 levels as biomarker for pancreatic ductal adenocarcinoma-associated malnutrition and muscle wasting. J Cachexia Sarcopenia Muscle. (2021) 12:704–16. doi: 10.1002/jcsm.12692, 33763996 PMC8200427

[67] McGovern J Grayston A Coates D Leadbitter S Hounat A Horgan PG . The relationship between the modified frailty index score (Mfi-5), malnutrition, body composition, systemic inflammation and short-term clinical outcomes in patients undergoing surgery for colorectal Cancer. BMC Geriatr. (2023) 23:9. doi: 10.1186/s12877-022-03703-2, 36609242 PMC9817261

[68] Luo T Huang C Zhou R Sun Y. Predicting complications in elderly patients undergoing Oral Cancer resection with free flap reconstruction in China: a retrospective cohort study using the modified frailty index and prognostic nutritional index. BMJ Open. (2024) 14:e085985. doi: 10.1136/bmjopen-2024-085985, 39730151 PMC11683887

[69] Huang W Wang C Wang Y Yu Z Wang S Yang J . Predicting malnutrition in gastric cancer patients using computed tomography(Ct) deep learning features and clinical data. Clin Nutr. (2024) 43:881–91. doi: 10.1016/j.clnu.2024.02.005 38377634

[70] Chawrylak K Homa-Mlak I Mazurek M Plecha E Brzozowska A Malecka-Massalska T . Mir-22-3p as a promising predictor of nutritional deficiencies in patients with head and neck cancer subjected to intensity-modulated radiation therapy. Sci Rep. (2024) 14:28120. doi: 10.1038/s41598-024-79641-3, 39548174 PMC11568149

